# Temperate Phages Acquire DNA from Defective Prophages by Relaxed Homologous Recombination: The Role of Rad52-Like Recombinases

**DOI:** 10.1371/journal.pgen.1004181

**Published:** 2014-03-06

**Authors:** Marianne De Paepe, Geoffrey Hutinet, Olivier Son, Jihane Amarir-Bouhram, Sophie Schbath, Marie-Agnès Petit

**Affiliations:** 1 INRA, UMR1319, Micalis, domaine de Vilvert, Jouy en Josas, France; 2 AgroParisTech, UMR1319, Micalis, domaine de Vilvert, Jouy en Josas, France; 3 INRA, UR1077, MIG, domaine de Vilvert, Jouy en Josas, France; Universidad de Sevilla, Spain

## Abstract

Bacteriophages (or phages) dominate the biosphere both numerically and in terms of genetic diversity. In particular, genomic comparisons suggest a remarkable level of horizontal gene transfer among temperate phages, favoring a high evolution rate. Molecular mechanisms of this pervasive mosaicism are mostly unknown. One hypothesis is that phage encoded recombinases are key players in these horizontal transfers, thanks to their high efficiency and low fidelity. Here, we associate two complementary *in vivo* assays and a bioinformatics analysis to address the role of phage encoded recombinases in genomic mosaicism. The first assay allowed determining the genetic determinants of mosaic formation between lambdoid phages and *Escherichia coli* prophage remnants. In the second assay, recombination was monitored between sequences on phage λ, and allowed to compare the performance of three different Rad52-like recombinases on the same substrate. We also addressed the importance of homologous recombination in phage evolution by a genomic comparison of 84 *E. coli* virulent and temperate phages or prophages. We demonstrate that mosaics are mainly generated by homology-driven mechanisms that tolerate high substrate divergence. We show that phage encoded Rad52-like recombinases act independently of RecA, and that they are relatively more efficient when the exchanged fragments are divergent. We also show that accessory phage genes *orf* and *rap* contribute to mosaicism. A bioinformatics analysis strengthens our experimental results by showing that homologous recombination left traces in temperate phage genomes at the borders of recently exchanged fragments. We found no evidence of exchanges between virulent and temperate phages of *E. coli*. Altogether, our results demonstrate that Rad52-like recombinases promote gene shuffling among temperate phages, accelerating their evolution. This mechanism may prove to be more general, as other mobile genetic elements such as ICE encode Rad52-like functions, and play an important role in bacterial evolution itself.

## Author Summary

Temperate bacteriophages (or phages) are bacterial viruses that, unlike virulent phages, have the ability to enter a prophage dormant state upon infection, in which they stably replicate with the bacterial genome. A majority of bacterial genomes contain multiple active or defective prophages, and numerous bacterial phenotypes are modified by these prophages, such as increased virulence. These mobile genetic elements are subject to high levels of genetic exchanges, through which new genes are constantly imported into bacterial genomes. Phage-encoded homologous recombination enzymes, or recombinases, are potentially key actors in phage genome shuffling. In this work, we show that gene acquisition in temperate phages is strongly dependent on the presence of sequence homology, but is highly tolerant to divergence. We report that gene exchanges are mainly catalyzed by recombinases found on temperate phages, and show that four different Rad52-like recombinases have a relaxed fidelity *in vivo*, compared to RecA. This high capacity of exchange speeds up evolution of phages, and indirectly also the evolution of bacteria.

## Introduction

Bacteriophages, or phages, the viruses that attack bacteria, have gained a renewed interest in the last decade with the emergence of antibiotic resistant bacteria. Despite their early discovery [1], [2] and the in-depth molecular and genetic characterization of few model phages, overall phage biology is still globally poorly understood comparatively to their ecological importance. Indeed, phages and related elements dominate the biosphere both numerically and in terms of genetic diversity. Despite more than 10^5^ genes already described in phage genomes, recent studies suggest that the majority of phage genes remains to be discovered [3]. The great genetic diversity of these viruses is due to their very ancient origin, their large population size and their high evolvability. Understanding evolvability of bacterial viruses will likely become a major issue with the prospective massive use of phages as alternatives to antibiotics. Notably, the mutation rate of viruses is much higher than that of cellular organisms [4], [5]. Horizontal gene transfer also plays a major role in virus evolution by creating new combinations of genetic material through the pairing and shuffling of related DNA sequences [6]–[8].

Initial observations of hybridizing segments between phage genomes by electron microscopy, and more recent genomic analyses, have revealed the pervasive mosaicism of temperate phage genomes [9]–[11]. Mosaicism refers to the patchwork character of phage genomes, which can be considered as unique combinations of exchangeable genomic segments [11]–[15]. Temperate phages, as opposed to lytic phages, have the ability to enter a prophage dormant state upon infection, in which they stably replicate with the bacterial genome. Nearly all bacterial genomes contain multiple active or defective prophages, the latter being unable to produce phage particles. In *Escherichia coli*, prophage genes can constitute up to 14% of the genome [16], and represent 41% of a 20 species pangenome [16]–[18]. Intergenomic rearrangements are thus facilitated for temperate phages by frequent encounters of different viruses inside the same bacterial host, for example between an invasive virus and a resident prophage.

While genome shuffling appears as a key driver of phage evolution, a quantitative description of mosaicism and analysis of its underlying molecular mechanisms are lacking. In particular, it is still debated whether phage genetic mosaicism is the product of recombination at sites of limited homology between genomes [19], [20], or the result of random, cut and paste, illegitimate recombination [11], [21]. In the latter hypothesis, the conservation of synteny would result from the counterselection of deleterious non-ordered gene combinations. Functions involved in homologous recombination (HR) have been extensively studied in *E. coli* and phage λ [22], [23]. In *E. coli*, the RecA recombinase is essential for catalyzing DNA exchanges between homologous molecules. However, the rate of successful RecA-dependent exchanges rapidly decreases with increasing sequence divergence [24], [25], suggesting that homologous exchanges should not happen between very divergent phage genomes. Phage genomes also encode recombinases catalyzing HR reactions, that have been classified into 3 super-families known as Rad51-, Rad52- and Gp2.5-like [26]. These recombinases are also found sporadically on non-bacterial viruses: archaeal proviruses encode Rad52-like genes [27] and Mimi and Herpes viruses encode a recombinase sharing homology with Gp2.5 [28]. Among the phage recombinases, the Rad52-like family is the largest and most diversified, and is itself subdivided into the Redβ, Erf and Sak groups. Among them, the λ recombinase Redβ is known to be efficient in the recombination of diverged sequences [19], [29], leading us to suggest that phage recombinases could be key actors in genomic shuffling of related phages.

The λ HR system, known as Red, is expressed during phage lytic development. Red consists of two functions encoded by the *redβ* and *redα* genes. The main activity of Redβ is to mediate single-strand DNA annealing between a Redβ-bound single-stranded region and a complementary sequence. λ Redα is a double-strand-specific exonuclease that generates single-strand DNA for Redβ annealing. Redβ can also promote recombination by a RecA-like strand-invasion mechanism, especially on short DNA sequences [30]. Two other genes in the λ nin genomic region, *orf* (former name *ninB*) and *rap* (former name *ninG*), were shown to facilitate RecA-dependent gene exchanges *in vivo* between strictly identical sequences [31]. The *orf* gene product is a mediator protein that participates in the loading of the bacterial RecA recombinase on SSB-coated DNA in the absence of the three bacterial proteins RecFOR [32]–[36]. The *rap* gene codes for a Holliday junction resolvase [35]. Orf and Rap have numerous homologs among temperate phages, and form ∼500 members families in Pfam. Interestingly, their distribution pattern among phage genomes is contrasted: among the 465 completely sequenced phage genomes collected in the ACLAME database, 191 encode a recombinase [26]. The presence of Orf is tightly associated with Recombinase+ genomes, as among the 55 phages encoding Orf, 49 also encode a recombinase, of the Rad52- or Rad51-like family. On the contrary, among the 180 genomes encoding Rap, only 100 are Recombinase+, as if Rap and phage recombinase occurrences were independent (MAP et al., to be published elsewhere). These distributions are suggestive of a more important role of Orf on phage recombinase activity. However, whether Orf or Rap stimulates Redβ mediated recombination is unknown at present.

Here, we study quantitatively the generation of mosaics between functional (i.e., infectious) temperate phages and defective prophages, and identify the genetic determinants of these exchanges. These assays reveal the preponderant role of Rad52-like phage recombination genes in exchanges involving short and diverged sequences. Moreover, a global analysis of mosaics between active temperate phages and defective prophages further reveals that these exchanges are commonplace in phage genome evolution.

## Results

### A recombination assay to measure formation of mosaics in phage λ

**Figure 1:**
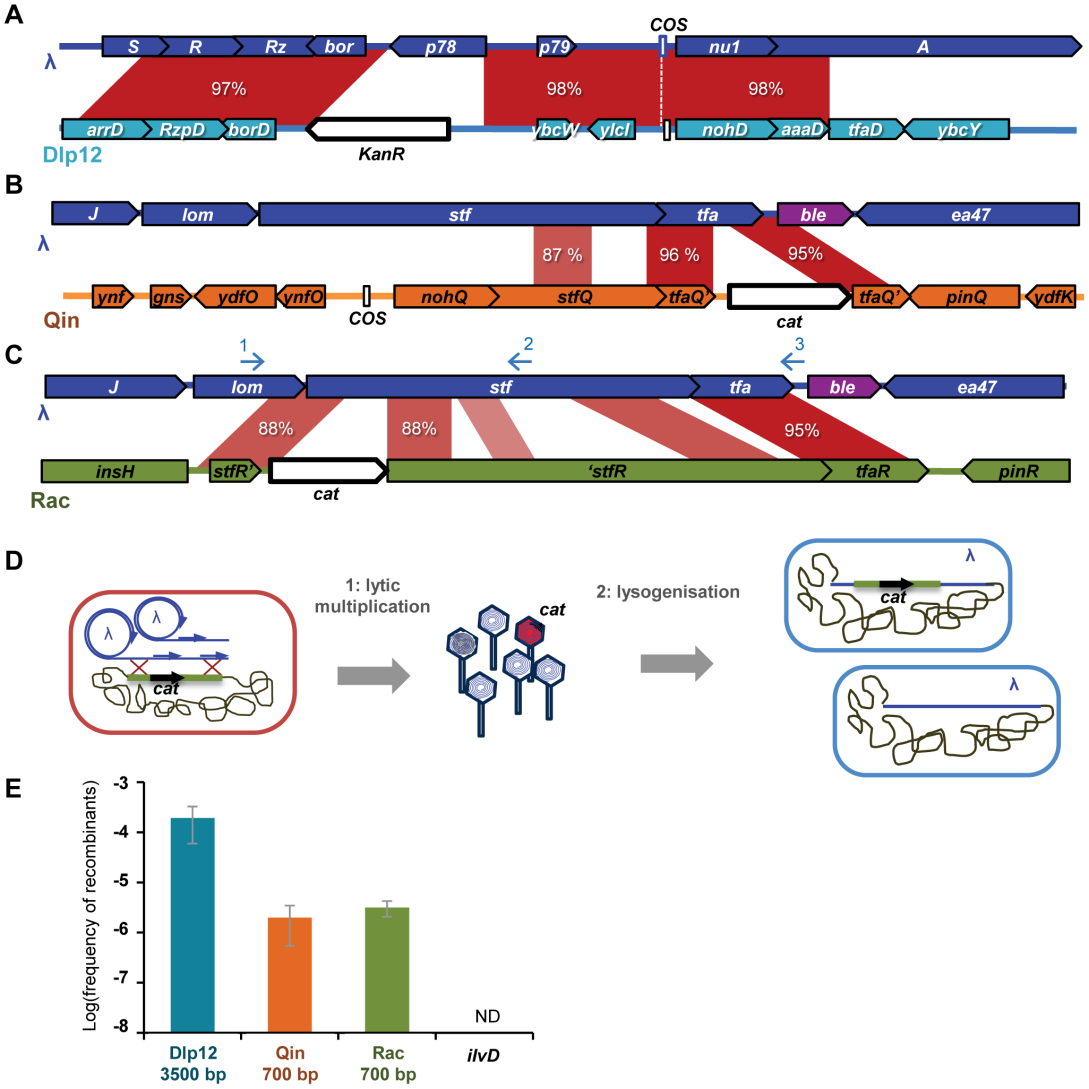
Recombinants between λ and defective prophages are formed during lytic cycle. A, B and C: Maps of the three regions of similarity between λ and MG1655 used in this study. Antibiotic resistance genes (white arrows) were inserted in the defective prophages, in the middle of the identity regions. *KanR* stands for the gene conferring resistance to kanamycine, the *cat* gene confers resistance to chloramphenicol. Identity region between λ and Dlp12 (A) spans across essential lysis genes (R and Rz) and terminase genes (A and nu1), separated by the *cos* site. Identity regions between λ and both Qin (B) and Rac (C) span across side tail fibers genes (*stf* and *tfa*). The *ble* gene, that confers resistance to phleomycine, was inserted between *tfa* and *ea47*, under the constitutive promoter P*sacB*. Blue arrows in C indicate the position of the 3 oligonucleotides used to sequence the recombinants. D: Schematic representation of the recombination assay: (1) λ phage is multiplied on a strain in which an antibiotic cassette has been inserted in a region of homology. (2) The phage produced is used to lysogenize a new strain. The total number of lysogenized bacteria is determined by their phleomycine resistance, while bacteria lyzogenised by a recombinant phage are also resistant to either chloramphenicol or kanamycin. E: Frequency of λ recombinants with Dlp12, Qin and Rac. Bp numbers indicate the size of the homology regions. Mean ± standard deviation of at least 3 independent recombination assays is indicated.

The aim of this first assay was to determine the extent and the mechanisms of genomic exchanges between an invasive infectious phage and defective prophages residing in the host chromosome. Homologous genomic regions between the MG1655 *E. coli* strain and λ phage were identified by a Blast search using relaxed parameters (same parameters as for the ANI analysis, see Fig. S6 legend). Twelve regions sharing over 70% mean identity on a stretch of at least 100 bp were found, all inside defective prophages. 3 such regions, differing in size and in the extent of identity, were selected for the experiments: i) a region in Dlp12 defective prophage, sharing 98% mean identity over 3353 bp with λ, ii) a region in Qin defective prophage sharing 96% mean identity over 657 bp, and iii) a region in Rac defective prophage sharing 88% mean identity over 703 bp, followed by another homology region nearby of 95% mean identity (Fig. 1 A, B & C). The length distribution of segments of perfect identity (segment without mismatch) is accordingly very different in the 3 regions (Fig. S1). Notably, the longest stretches of perfect identity are 298, 115 and 49 bp for Dlp12, Qin and Rac respectively. An antibiotic resistance gene, conferring resistance to either chloramphenicol (*cat*) or kanamycin (*kanR*), was introduced in the middle of each selected region (Fig. 1 and Materials and Methods). As a control, we used a strain with the *cat* gene into *ilvD*, which has no homology with λ and whose deletion does not impact phage growth.

Defective prophages can reportedly excise and even replicate in different strain backgrounds under certain conditions, notably during phage infection [31], [37], [38]. We thus verified by PCR that the 3 studied defective prophages do not excise during infection by λ. Moreover, as Rac has an active but normally repressed replication origin, we checked by semi-quantitative PCR whether its replication was induced upon λ infection. We found no evidence of such a phenomenon (Fig. S3).

Recombination between λ and the marked defective prophages can result in the integration of *cat* or *kanR* antibiotic resistance gene into λ (Fig. 1E). As this does not produce a directly detectable phage phenotype, *E. coli* cells were further lysogenized with the resulting phage, and the proportion of cells lysogenized by WT or antibiotic-resistant recombinant phages was determined by plating on selective antibiotic media (Fig. 1D and Materials and Methods). To be able to detect all lysogenized bacteria, we used a λ strain marked with the phleomycine resistance gene *ble* (Urλ*ble* strain, Table S1 and Materials and Methods). This recombination assay enables the estimation of the horizontal gene transfer rate into the λ genome when it infects its host.

### Mosaicism is produced by homologous recombination

**Figure 2:**
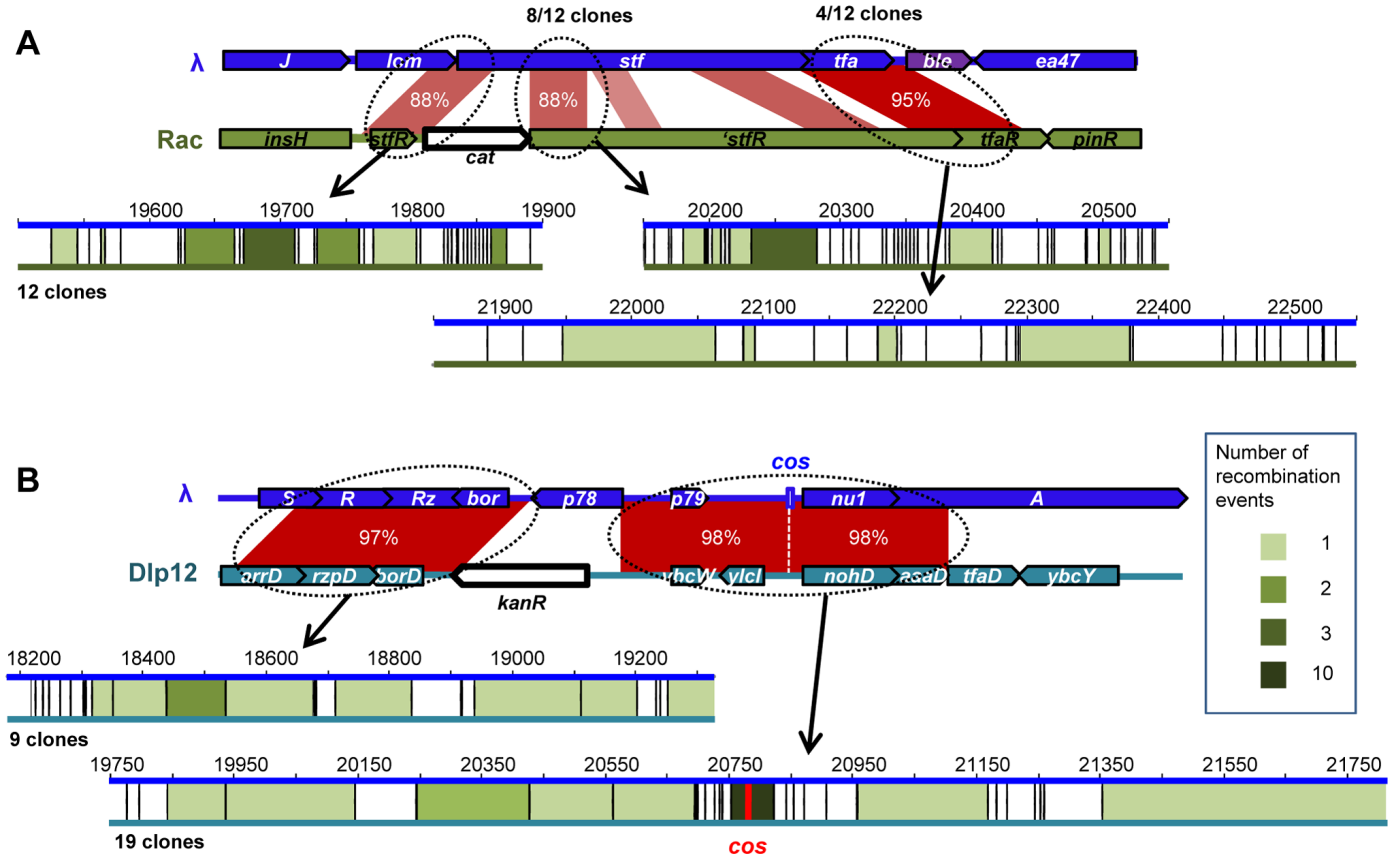
Position of recombination events in homology regions between λ and defective prophages. A: Positions of recombination events between Rac and λ for 12 sequenced clones. Mismatches in similarity regions between rac and λ are represented by vertical black bars. Intervals between two mismatches in which sequencing revealed that recombination occurred are colored in green, intensity representing the number of recombination events (see legend). B: Positions of recombination events between Dlp12 and λ. Nine and 19 clones were sequenced on the left and right sides of *cat* gene, respectively. Half of the recombination events scored occurred at direct proximity of the *cos* site.

λ recombinants with Rac and Qin were observed at frequencies around 2×10^−6^, and were a hundred fold more numerous with Dlp12 (Fig. 1E). Interestingly, the frequencies of recombinants were similar for Rac and Qin despite different extent of identity on comparable lengths (Fig. 1 and S1). In contrast, the recombinant frequency with the control chromosomal gene *ilvD*, presenting no homology with λ genome, was below our detection threshold of 5×10^−9^. PCR analysis on 20 λ recombinants within each of the 3 loci showed that all had incorporated the resistance gene at the expected position for an homologous exchange. 12 to 19 were sequenced to confirm that homologous recombination occurred in the targeted region and to identify the exact junctions (Fig. 2). Among recombinants with Rac, the majority resulted from recombination events within the adjacent 88% identity regions, but one third had recombined on the right in the nearby 95% homology region in *tfa*.

HR with Rac and Qin leads to the inactivation of λ side tail fibers genes (*stf* and *tfa*), which reportedly improves phage growth compared to the parent in a soft-agar overlay [39]. To evaluate if this increases the recombinant frequency by favoring their growth, we performed the same recombination assay with the sequenced λPaPa strain of λ, mutated in its *stf* gene [40]. The frequency of λPaPa recombinants with Rac was not significantly different from that with Urλ (p = 0.18, Student T-test), ruling out the possibility that the advantage of side tail fiber inactivation distorts our conclusions. This can be explained by the low recombination rate: most recombinants statistically arise only during the last (and third) cycle of phage growth, as phage engaging in the third lytic cycle are 10,000 more numerous than those involved in the first one. Sequencing of 12 λ-Rac recombinants showed that they all correspond to different hybrids (Fig. 2A), confirming the absence of amplification and also indicating the absence of a recombination hotspot.

The high recombination frequency with Dlp12 is expected for two reasons. First, Dlp12 shares the largest region of homology with λ (3353 bp), and secondly, the region of homology contains the λ *cos* site (Fig. 1A). Double-strand DNA breaks at *cos* sites, created for genome encapsidation, stimulate recombination on its left side, the right side bound by the terminase being protected [41]. Double-strand breaks in both λ and Dlp12 might thus stimulate the recombination in this region. Sequencing of 19 λ-Dlp12 recombinants indeed revealed a high proportion of junctions in the immediate proximity to *cos* (10 out of 19 clones, Fig. 2B). 3 out of 19 junctions were nevertheless found on the right side of *cos*, indicating that double-strand breaks at *cos* improve but are not necessary for recombination events. Interestingly, all tested λ-Dlp12 recombinant phages were active, including those that had replaced the essential λ genes R and Rz (lysis) or *nu1* (terminase) by those of Dlp12, indicating that these Dlp12 genes are functional in λ.

### Recombination genes involved in mosaicism

We have shown that genetic exchanges with λ are driven by the presence of homologous regions. We next questioned the respective roles on these exchanges of the bacterial and phage HR genes, *recA* and *redβ*, and also of two other λ recombination genes, *orf* and *rap*. As *redβ* and *redα* λ mutants have a reduced burst size (Fig. S2), conditions of phage growth on the marked strains were adapted to ensure that the same number of phage generations was realized (see Materials and Methods).

**Figure 3:**
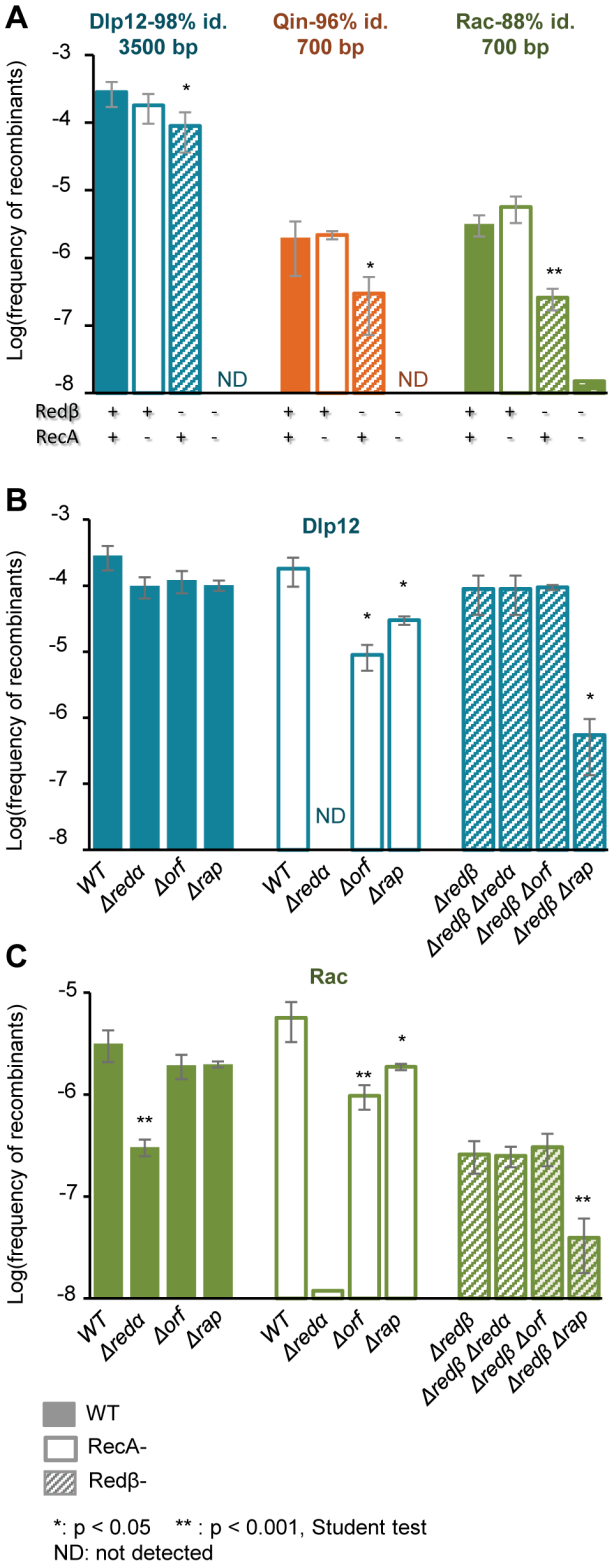
Recombinants between λ and defective prophages are formed preferentially by the Red-pathway, especially when sequences are short or diverged. A: Frequency of recombinants with Dlp12, Qin and Rac, as a function of Redβ and RecA presence (indicated below the bars). Both recombinases are able to catalyze the exchanges, but Redβ participation is more pronounced, especially on short and diverged sequences. When both recombinases are absent, almost no recombinants are obtained. B: Role of *redα*, *orf* and *rap* genes on the exchanges in the large region of high homology between λ and Dlp12. Analysis is performed in the presence of both RecA and Redβ (full bars), and also with only Redβ (empty bars), or RecA (stripped bars). Phage genotypes are shown below the bars. C: Same genetic analysis on the short (700 bp) and more diverged region of homology between λ and Rac. Mean ± standard deviation of at least 3 independent recombination assays are indicated.

The frequency of recombination with the large, quasi-identical segment of Dlp12 (3.3 kb, 98% identity) was not affected by a single *recA* deletion, and only 3-fold by a *redβ* deletion (Fig. 3A, blue bars). However, when both recombinase genes were deleted, exchanges were completely abolished. This shows that both RecA and Redβ produce recombinants independently of one another, which is indicative of redundant pathways. Within Qin and especially Rac regions, showing more divergence with λ, single *recA* deletion had similarly no effect on recombination frequencies. The single *redβ* deletion however had a pronounced effect on the recombination frequencies (6- and 13- fold respectively, Fig. 3A, orange and green bars), indicating that most recombination events are formed by Redβ when homology is reduced, reflecting a lowest activity of RecA on these substrates. The residual recombinants formed in the absence of Redβ almost completely disappeared with the double deletion of *recA* and *redβ*. Only 2 recombinant clones were obtained in the *recA redβ* double mutant, with Rac, and were found by PCR to result from homologous recombination. They might have originated from the expression of the *recET* recombination genes of Rac. Normally the *recET* genes are completely repressed in *E. coli* MG1655, even upon λ infection [42], and thus cannot promote recombination, but rare *sbcA* mutations [43] or incorporation in λ (the so-called λ-rev genotype, [44]) can activate them.

### Role of other phage encoded recombination promoting genes

The activities of recombinases are stimulated by numerous host or phage-encoded cofactors, which either prepare the substrate for recombination or act at latter stages to resolve the DNA heteroduplex structure. The main partner of Redβ is the double-strand-specific exonuclease Redα that transforms double-strand DNA into single-strand DNA, the substrate for Redβ. Redα is dispensable for Redβ recombination if the DNA substrate is initially in a single-strand form [45]. Here, deletion of *redα* gene had the same effect as the *redβ* deletion, and basically disabled Red-mediated recombination in Dlp12 and Rac regions (Fig. 3B&C). This finding reveals a need for single-strand DNA formation for Red-mediated recombination in our assay.

We then tested the effects of other recombinase helper proteins on recombinant frequencies. λ encodes two such proteins, Orf and Rap, which belong to protein families highly prevalent in phage genomes (respectively 55 and 180 homologues in 465 phage genomes, our unpublished work). Orf and Rap participate in RecA-dependent recombination by supplying a function equivalent to bacterial encoded recombination cofactors, respectively RecFOR and Ruv resolvase [36], [46]. Here we asked whether they could also participate in Red recombination, and whether their role was more pronounced on diverged sequences than on easy to recombine long sequences. Single deletion of *orf* or *rap* resulted in a not significant 3-fold reduction of λ recombination with Dlp12 (Fig. 3B, first set of data). However, in a *recA* strain, in which all gene exchanges are Red-mediated, recombination was reduced by 10-fold in the Δ*orf* mutant, and by significant 3-fold in the Δ*rap* mutant. This result reveals that Orf and to some extent Rap participate in Red-mediated recombination. Finally, in the genetic context where RecA mediates all gene exchanges, the *orf* deletion had no effect, while the *rap* deletion decreased recombinant frequency by 300-fold, as expected from an earlier report [47]. The same genetic analysis in the Rac region assay revealed the same dependencies (Fig. 3C). In conclusion, Orf and to a lesser extent Rap facilitate homologous recombination with λ when Redβ is the only recombinase, with no indication of an increased role when DNA sequences are more divergent.

### Homologous recombination-dependent gene shuffling also occurs with phage Φ80

In order to determine the generality of our observations with λ, we performed similar recombination assays with the lambdoid phage Φ80. Its genome homology with λ is mostly clustered in the capsid region, while the rest of the genome is highly divergent with only few segments of homology [48]. Φ80 encodes a putative recombinase from the Rad52 family, hereafter named RecT_Φ80_, that shares 32% identity at the amino acid level with Redβ.

**Figure 4:**
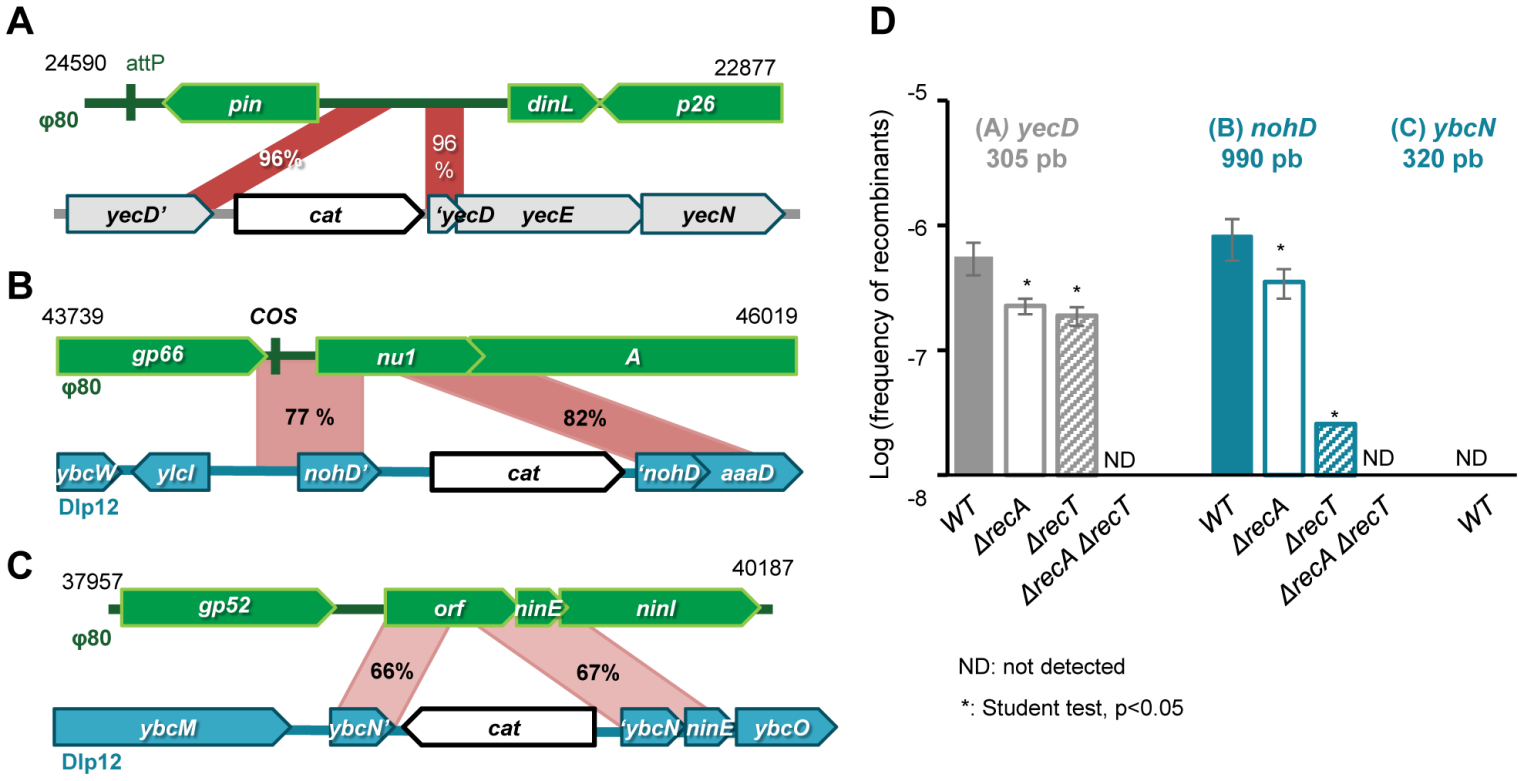
Formation of Φ80 hybrids also depend on phage recombinase. A: map of the 300 bp region of 96% identity between the Φ80 region near attP site and *E. coli* gene *yecD*. B: Map of the 980 bp homology region between Dlp12 and Φ80, spanning through the essential terminase genes *A* and *nu1*. C: Highly divergent 500 bp homology region between the non-essential nin region of Φ80 and Dlp12. D: Frequency of recombinants for each homology region, as a function of Redβ and RecA (genotypes are indicated below the bars). Mean ± standard deviation of at least 3 independent recombination assays are indicated.

As previously described with λ, we detected by Blast search 9 regions of homology between Φ80 and *E. coli* MG1655 genomes, and selected 3 of them for the recombination assay: i) the 3′ part of the bacterial core gene *yecD*, of unknown function, presenting 96% mean identity over 300 bp, ii) in the *nohD* region of the defective prophage Dlp12, sharing 79% identity over 980 bp with the terminase genes *nu1* and *A*, and iii) within the *nin* region of Dlp12, sharing 67% identity over 500 bp (Fig. 4 A, B, C). For each region, the longest stretch of perfect identity is 116, 41 and 11 bp for *yecD*, *nohD* and *nin*, respectively (Fig. S1B). Homology regions were labeled with the *cat* gene, and Φ80 genome was marked with the *ble* gene. Φ80 phage was then propagated on the modified *E. coli* strains and the number of recombinant phages was scored (Fig. 4D).

Recombinants were produced at a frequency of 6×10^−7^ per phage with the *yecD* locus and 8×10^−7^ with the *nohD* locus of Dlp12 (Fig. 4D). In the last case, recombination disrupts the essential terminase genes *nu1* and *A*, but this does not prevent encapsidation of recombinant Φ80 genomes, due to the terminases encoded by the other copies of Φ80. However, the recombinants formed will not give lytic progeny upon infection of a new host, ensuring that the recombinants counted in the assay are exclusively those formed during the last lytic cycle. As discussed above, the presence of the *cos* site within the homology region probably increases the production of recombinants. Within the last region, sharing only 67% identity over 500 bp with λ, the recombination frequency was below 1×10^−8^, our detection threshold (*ybcN* locus, Fig. 3C). PCR analysis revealed that the 12 recombinants scored had incorporated the resistance gene at the locus expected for homologous exchange.

As with λ, we then determined the respective role of homologous recombination enzymes RecA and RecT_Φ80_ in these genetic exchanges. *recA* deletion slightly diminishes recombinant frequency by 2-fold on both loci, whereas *recT_Φ80_* deletion has a pronounced effect on the more diverged sequences (Fig. 3C).

These results show that homology-dependent mosaic formation driven by phage encoded recombinases is not restricted to λ and may be a general event among temperate phages encoding their own homologous recombination functions. Exchanges occurred even when the resulting recombinant phages were no longer viable as a lytic phage, and appeared to be only limited by the degree and length of sequence homology.

### Like Redβ and RecT_Φ80_, Erf_D3_ and RecT_Rac_ recombinases are efficient on diverged sequences

**Figure 5:**
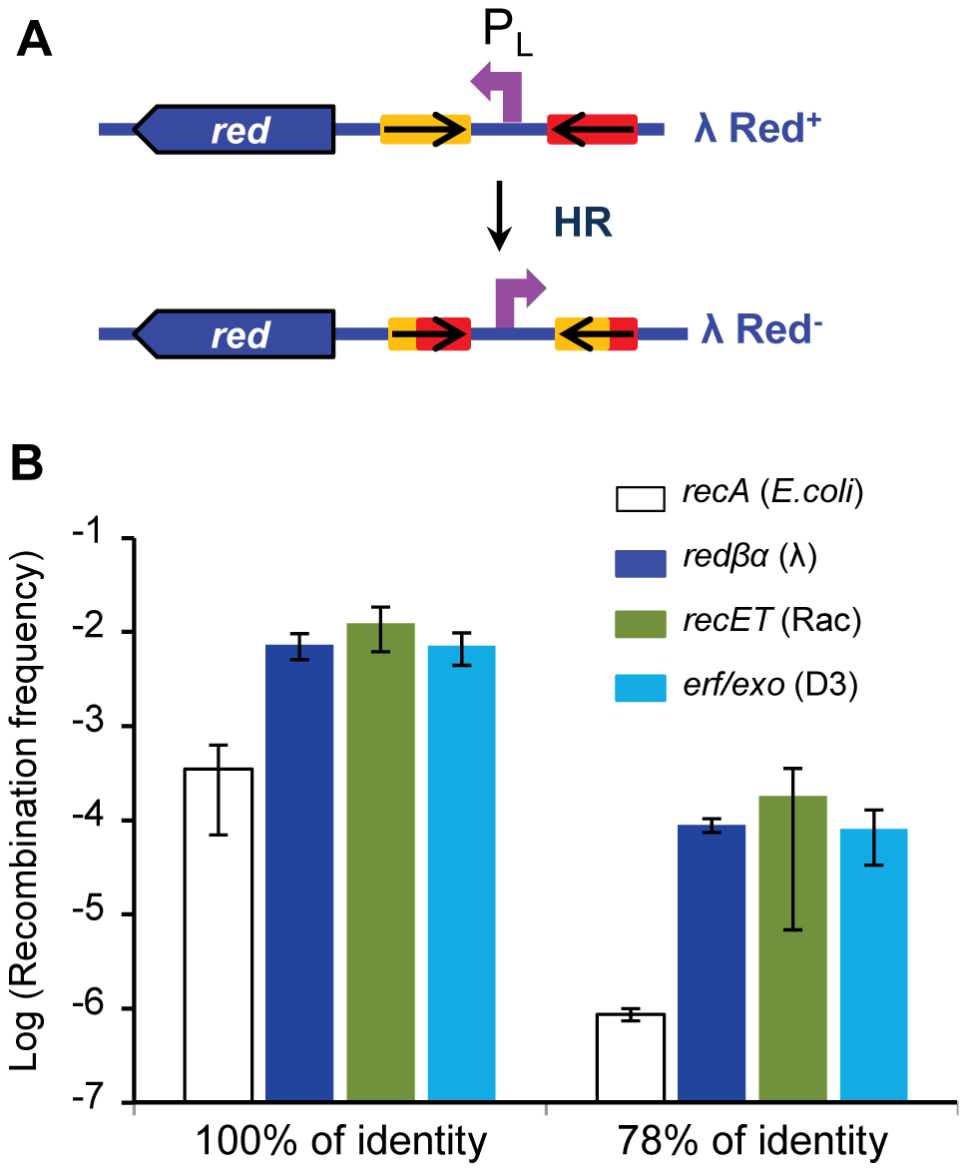
High efficiency of recombination of two other Rad52 recombinases. A: Principle of the assay: two 800 bp homologous sequences (*oxa* genes, as in [19]), inversely repeated, represented by the red and yellow rectangles, are inserted so as to flank the P*_L_* promoter in the λ genome. Inverted sequences are either 100% or 78% identical. When homologous recombination occurs between the repeated sequences, the P*_L_* promoter is inverted, which leads to a phenotypic switch, because the *red* and *gam* genes are no longer expressed (see Materials and Methods). B: Recombination frequencies scored with three different pairs of phage recombinase/exonuclease, and compared to RecA pathway. The *recET* (from Rac prophage) and *erf/exo* (from phage PA73) genes were substituted in place of the λ *red* genes. Values shown for *redαβ* and *recA* pathways are those reported in [19]. Mean ± standard deviation of at least 3 independent recombination assays are indicated.

The capacities of Rad52-like phage encoded recombinases and cofactors were further explored with an intra-bacteriophage recombination assay, which enables the comparison of recombinase activities on the same DNA substrate. We monitored homologous recombination within the λ genome, between two inverted 800 bp sequences introduced on each side of the P_L_ promoter (described in Fig. 5A and [19]). Briefly, inversion of the P_L_ promoter leads to a detectable phenotypic switch, as it prevents transcription of the *red* and *gam* genes, enabling growth on a P2-lysogen, contrary to the non-inverted phage. Vice versa, inverted P_L_ Red^−^ Gam^−^ phage cannot grow on a *recA* strain, in contrast to the non-inverted wild-type phage. Two different recombination cassettes were used, the inverted sequences being either strictly identical, or 78% identical. The switch was measured in the two directions, and in all cases, the recombination assay was performed by growing phages on a restrictive host for the multiplication of the recombinants, ensuring that the recombinants are produced only during the last generation.

In this study, λ *redβ* and *redα* genes were replaced by other pairs of recombinase genes and their respective associated exonuclease. The first pair is *recT* and *recE* (fragment coding for the Cter part of the protein), from the defective prophage Rac. The second pair is the predicted Erf family recombinase gene *erf*, with its associated predicted exonuclease gene *exo*, from D3 phage infecting *Pseudomonas aeruginosa* [49]. Resulting phages were grown to confluence on a bacterial lawn, and the frequency of recombinants was measured by differential platings. On both the 100% and 78% identical substrates, efficiency of recombination mediated by the RecT/RecE_Rac_ and Erf/Exo_D3_ pairs was similar to that mediated by λ Red proteins (Fig. 5B). First, this demonstrates that the D3 *erf* gene product is indeed a recombinase, and as efficient as Redβ and RecT_Rac_. For all 3 recombinase/exonuclease pairs, recombination between identical or diverged sequences was respectively 20- and 100-fold higher than in the RecA pathway.

This result extends our previous observation with λ and Φ80, and suggests that during the lytic cycle, the high efficiency and low fidelity of phage-dependent recombination might be a general phenomenon that promotes horizontal gene transfer into phage genomes.

### Genomic analysis reveals abundant mosaics among and between temperate and defective phage genomes, but none between virulent and temperate genomes

We thus attempted to quantify the traces that such exchanges might have left on phage genomes on an evolutionary time scale by analyzing a collection of *E. coli* phages (Table S4). In particular, we examined whether traces of HR events could be detected at the boundaries of recently exchanged DNA fragments. In a previous study of mosaics formed between temperate lambdoid phages [19], we showed that half of the mosaics, defined by Blast hits with more than 90% identity, were flanked by at least one region sufficiently similar to be an indication of a region of homology preexistent to the recent exchange identified by the Blast hit. These above background homology regions were hereafter named “HR traces”. This study was extended and refined here, so as to include more genomes from temperate (24 genomes), defective (34 genomes) or virulent (26 genomes) *E. coli* phages.

**Figure 6:**
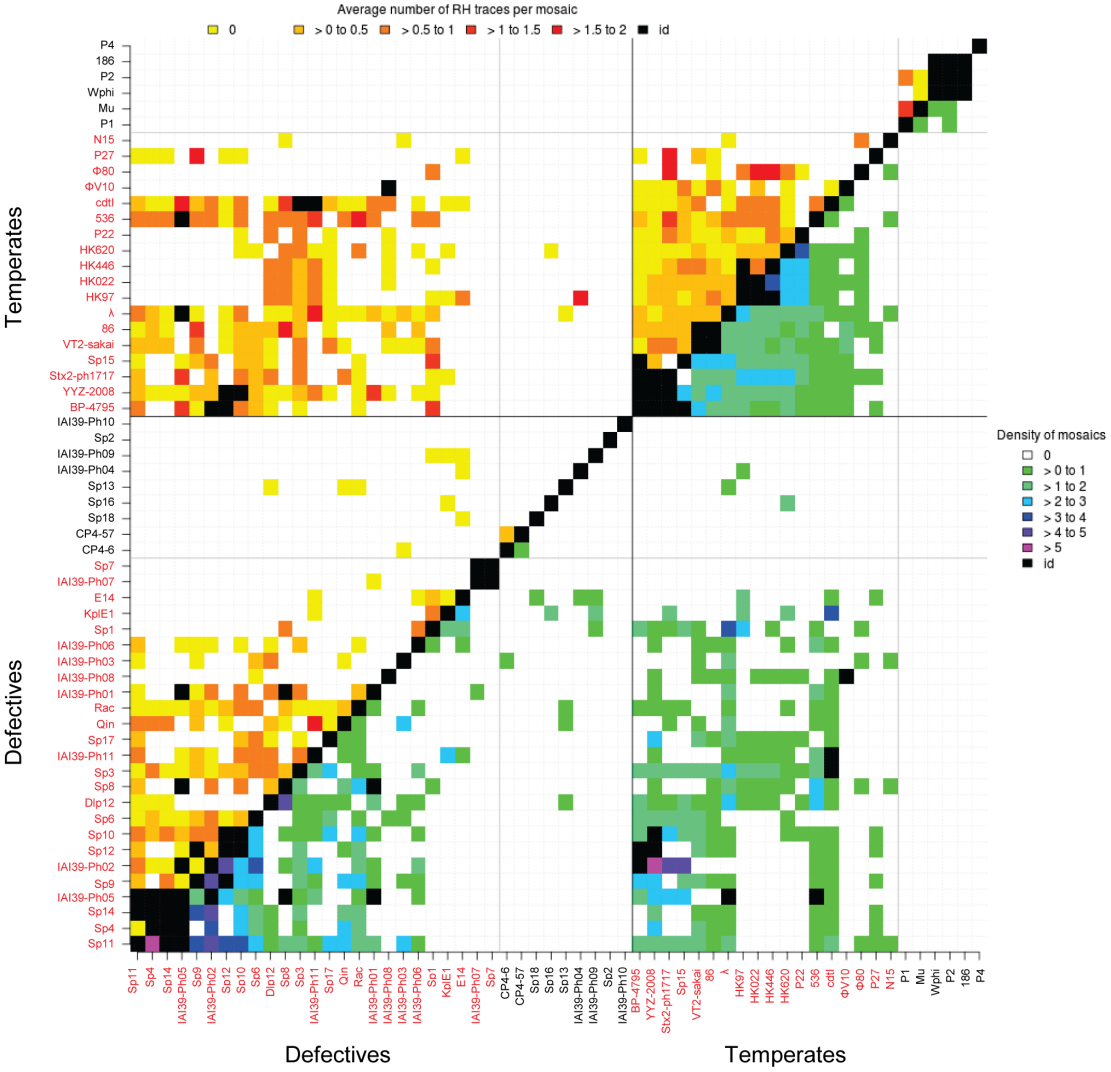
Heat map of mosaic characteristics in temperate and defective phages. Density of mosaics found among pairs of phages (lower part of the diagonal, purple to green colours), and average number of traces of recombination detected at the boundary of mosaics (higher part, yellow to red colours). The density is the number of mosaics per 10 kb of phage genome. Names of λ-like phages are in red.

Results are summarized in Table 1. We detected a large number of recently exchanged genomic segments among temperate and defective phages (between 0.8 and 2.4 mosaics per genome pair). No mosaic was found between virulent and temperate or defective phages. We also found a few mosaics between virulent phages (0.06 per genome pair, Table 1). Interestingly, among temperate and defective phages, the density of mosaics, *i.e.* the numbers of mosaics per 10 kb, was similar (Fig. 6, lower part of the diagonal), suggesting that exchanges do occur at similar frequencies among defective and functional temperate phages. Among temperate phages, two sub-groups not sharing mosaics were found: the larger one contained lambdoid phages (i.e. λ-like, ε15-like and P22-like), the smaller group contained P2-like, P1 and Mu-like phages. Among pairs involving defective phages, exchanges between these two groups are found, probably because non functional gene combinations are not counterselected in defective prophages. Interestingly, the size distribution of mosaics indicates that small gene fragments are much more often exchanged than complete functional modules (Fig. S5).

**Table 1:** Data collected on mosaics, by categories of genome pairs compared.

Phages pairs	T-T*	T-D	D-D	V-V	V- others
N genome pairs analysed (p)	266	807	549	289	1508
N of Blast matches	711	895	670	16	0
% of IS matches	9.7	26.7	33.6	0.0	0
N of mosaics analyzed for HR traces (m)	635	631	430	16	0
N mosaics/genome pair (m/p)	2.39	0.78	0.78	0.06	0
Median mosaic length (bp)	404	494	458	131	0
Median mosaic id%	96.1	94.8	95.1	91.5	0
N of mosaics without HR traces	432	411	298	5	0
N with 1 trace of HR	169	171	106	6	0
N with 2 traces of HR	34	49	26	5	0
P (≥1 trace of HR at random)	<10^−16^	<10^−16^	<10^−16^	1.8 10^−14^	

*T, temperate, D, defective, V, virulent.

**Figure 7:**
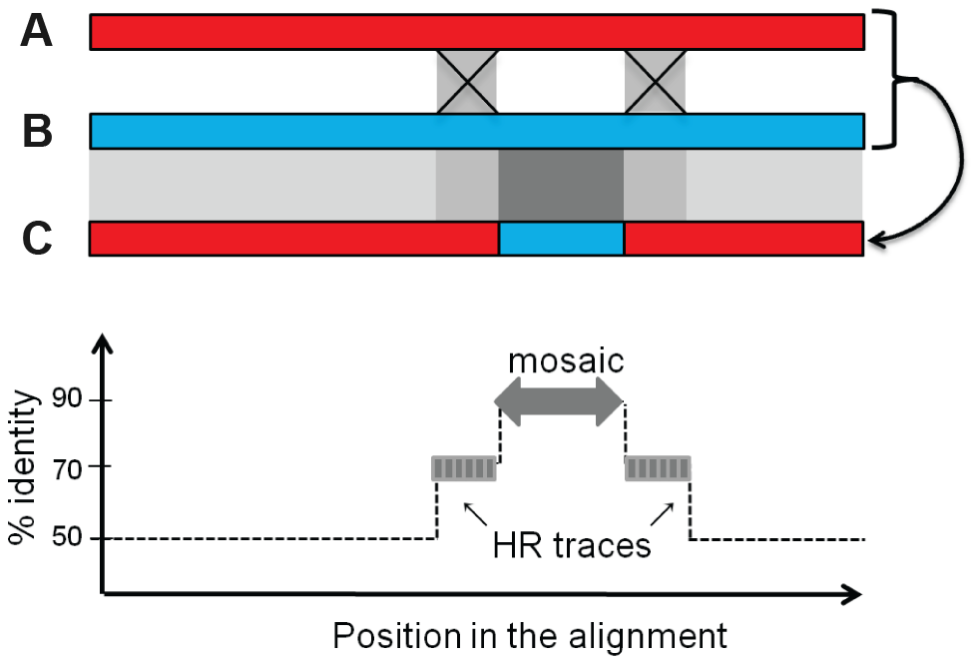
Rationale for the bio-informatics detection of mosaics. Upper panel: two ancestral phages A and B recombine across two regions of partial identity (light grey squares). As a consequence, the new piece of DNA in the recombined phage C, when compared to its parent B providing the mosaic, exhibits a 100% identity region (dark grey square), flanked by the two partially identical sequences (light grey), above the background level *b* of low identity shared by B and C. Upon alignment of genome C with B around the mosaic (lower panel), if the regions flanking the mosaic have a percentage of identity above the background level of identity (≥*b*+10%), they will be counted as traces of homologous recombination (HR trace).

We next investigated whether the mosaics had HR traces, i.e. flanking regions that have a lower mean identity than the mosaic but a higher than expected identity (see Figure 7 and Text S1 for the principle of analysis). Briefly, HR traces were detected by complete realignment of the mosaic flanking regions, and identification of above-background levels of nucleotide identity. We found regions suggestive of HR for 32% of the mosaics occurring among temperate phages, 35% of those formed between defective and temperate phages, and 31% for the inter-defective mosaics (Fig. 6, upper side of the diagonal, and Table 1). In most cases, only one, rather than two HR traces were found flanking the mosaics. This may be explained by other mechanisms than HR to generate exchanges but also by successive rounds of exchanges, involving different phage pairs, as illustrated in Fig. S7.

## Discussion

It has been known for a long time that related phages can form recombinant hybrids when infecting simultaneously the same bacterial cell, or by recombination with integrated prophages [6], [50]–[52]. λ defective mutants in particular can be rescued by genes present on prophages [31]. Here we quantified for the first time these exchanges between phage genomes, and helped to precise the rules that dictate phage mosaicism, demonstrating that they are dependent on homologous recombination enzymes, with a preponderant role for the phage-encoded recombinases when sequences are diverged. Our results suggest that illegitimate recombination is probably much less frequent during phage genome evolution. This high level of exchanges among phage sequences is to be contrasted with the barrier observed against similar exchanges during bacterial recombination: on *E. coli* plasmids, recombination involving 4% diverged sequences is reduced by 10,000-fold compared to identical sequences [53], whereas during the lytic cycle, recombination between 12% diverged sequences is reduced only by a 100-fold compared to identical sequences (Fig. 5B).

### Mosaic formation is driven by Rad52-like phage recombinases

We demonstrate that phage encoded Rad52-like recombinases play a primordial role in recombining regions sharing only short stretches of homology. Interestingly, on these substrates, all four Rad52-like recombinases that we tested (Redβ, RecT_Rac_, Erf_D3_ and RecT_Φ80_) had a higher activity than RecA during λ replication. These results strongly support the view that phage Rad52-like recombinases, predominant among *E. coli* lambdoid phages [54], play a crucial role in genomic shuffling. Interestingly, a recent bioinformatic study showed that the level of mosaicism is higher for phages encoding a recombinase than for others [54], supporting this hypothesis. Whether the same holds true for the two other large families of recombinases encountered in phages (Gp2.5 and Rad51, [26]) remains to be investigated experimentally. *Staphylococcus aureus* temperate phages exhibit a large level of mosaicism [19], and encode indifferently all three types of recombinases, which suggests that the two other families might also have the same property of relaxed fidelity. Further work aiming at comparing these recombinases side by side will allow addressing this point in detail.

The high efficiencies of Rad52-like recombinases on diverged sequences could be due to their higher concentration or activity, compared to RecA, during phage infection. However, on highly homologous sequences, RecA or phage recombination pathways result in a similar yield of recombinants (Fig. 3A and 4D). The 10–20 fold higher efficiency of Redβ or RecT_Φ80_ compared to RecA pathway is unveiled only on more divergent sequences. It could result from activity on shorter perfect sequence identity segments to recombine as compared to RecA. The Minimal Efficient Pairing Segment (MEPS), the minimal size of exact pairing required for efficient exchange *in vivo*, has indeed been found to be 31–34 bp for RecA [24] and only 23–27 for Redβ [24], [55]. Our *in vivo* assays involve sequences that do not have regularly spaced mismatches, so that slight changes in the number of segments above MEPS size may have drastic effects. Interestingly, Li and collaborators have recently reported that recombineering with regularly spaced, diverged oligonucleotides is effective up to 1 mismatch every 5 nucleotide, which corresponds to a mean divergence of 17% [29], and fits nicely our observations. The low sensibility to divergence of phage recombinases could also result from different sensitivities to methyl-directed mismatch repair (MMR), comprising MutSLH proteins. Indeed, the high fidelity of homologous recombination is caused not only by the intrinsic properties of the enzymes, but also by MMR inhibition of exchange if mismatches are present in recombination intermediates [56]. Classically, defects in MMR provoke a 100-fold increase of HR, either in RecA [57] or Redβ pathways [58]. However HR is less affected by the MMR during the λ lytic cycle: RecA-dependent recombination is enhanced by only 2 to 8 fold in a *mutS* background, depending on the level of divergence, and Redβ-dependent recombination is not affected at all [19]. Whether mismatch repair is titrated or inhibited during the phage lytic cycle is an open question that deserves further inquiry.

In λ, *red* mutants have a 5-fold reduced burst size (Fig. S2), which at present remains unexplained (see [59] for a review discussing this point). Our observation that RecA substitutes completely for Red for HR on identical sequences seems to exclude that the loss of viability of *red* mutants is due to a strict HR defect. RecBCD pathway is inactive in λ infected cells as λ lacks Chi sites and moreover express a RecBCD inhibitor. Further, we could not detect any synergy of the *red* and *recA* mutations on plaque sizes (Fig. S4). Whether Red impacts replication or any other stage of the phage cycle remains to be investigated, as well as why Φ80 recombinase does not impact Φ80 plaque size.

Biochemical evidence suggests that Sak and Sak3, two Rad52-like recombinases of lactococcal phages, act as cofactors of RecA [60], [61]. Some *in vivo* work also pointed to such a role for Redβ on non-replicating λ genomes [62]. In the present work, the phage and host recombinases appear rather to work independently from each other, and redundantly on highly similar sequences.

The RecA cofactor function of phage-encoded Rad52-like proteins may therefore be a minor activity *in vivo*. This situation is contrasted with the yeast-encoded Rad52 protein, which is essentially known for its Rad51 cofactor activity. However, Rad52 also performs some repair reactions in a Rad51 independent way [63]. Whether these Rad52 activities are tolerant to diverged DNA is unknown at present. Interestingly, another kind of mobile genetic elements that present genomic mosaicism, the integrative conjugative elements of the SXT/R391 family, has been reported to encode a recombinase of the Rad52 family (named s065) [64]. It was found to act in the formation of hybrid ICEs, independently of RecA. The assay involved 95–97% identical sequences, and RecA was the dominant pathway. Whether s065 becomes dominant when recombination involves more diverged sequences, remains to be investigated.

### Contribution of accessory proteins Orf and Rap to recombination

Interestingly, both Orf and Rap proteins have numerous homologs among temperate phages, and the presence of Orf is strongly associated with the presence of a recombinase. Notably, Φ80 possesses homologs of both *orf* (*gp53*) and *rap*. Rap (recombination adept with plasmid) increases RecA-dependent recombination between λ and a plasmid sharing perfect identity by a 100-fold [47]. In line with this result, we found that in the RecA pathway, *rap* deletion results in a 500-fold decrease in recombination with Dlp12. With the Rac substrate, where the activity of the RecA pathway is minor, *rap* deletion results in a further 10-fold decrease, again underlining the importance of *rap* in this pathway. For Redβ-dependent recombination, the decreases due to *rap* deletion were only 3 and 6-fold for Dlp12 and Rac substrates, respectively. This is probably related to the fact that the Rap substrates, Holliday junctions, are not necessarily formed by Redβ Indeed, Redβ principally recombines by a strand assimilation mechanism, which consists in the single-strand annealing between Redβ-bound single-strand DNA and the lagging strand template of a replication fork, a reaction that does not need a resolution step [65]–[68]. On the contrary, RecA catalyses mainly strand invasion reactions, which generate Holliday junctions [69], [70]. The Rap protein acts essentially independently from Redβ, a conclusion which agrees nicely with the independent genomic distribution of these two genes.

Orf is involved in displacing of SSB from single-stranded DNA, which facilitates RecA binding. We found that *orf* deletion decreases Redβ dependent recombination by 10-fold, and does not affect the RecA pathway. This is in line with the strong association observed between *orf* and recombinase genes in phage genomes. This Orf effect might reflect the proportion of cases where Redβ enters in competition with SSB. As Redβ loads onto single-strand DNA immediately after Redα, the two proteins being supposedly in interaction, the Orf activity to remove SSB should not be essential, as SSB might not be present on most of the substrates recombined by Redβ.

### Quantification of mosaics and traces of homologous recombination in phage genomes

The presence of all these recombination genes in phage genomes influences their long term evolution. Indeed, here we add evidences that HR leaves frequent traces among phage genomes. We found recently formed mosaics - defined as sequences sharing more than 90% identity - between temperate and defective genomes, but also a few ones among virulent phages. Interestingly, recent gene exchanges were identified only inside the lambda-like group or the P1-P2-Mu group, but not between each group. Traces of homology flanking the mosaic were detected in ∼30% of the mosaics between temperate phages and/or defective prophages. Whether the remaining 70% mosaics were formed by HR but have shorter or masked traces (Fig. S7), or were created by non-homologous recombination remains an open question.

Remarkably, no recent mosaics were found between temperate and virulent phages infecting *E. coli*. In fact, a well documented case of ancient horizontal gene transfer among a large group of unrelated temperate and virulent phages exists, and concerns the side tail fiber (*stf*) genes [71]–[73]. However, this case is very specific, as these genetic exchanges are most probably driven by *stf* associated phage invertases that catalyze site-specific recombination [74]. The very low potentiality of virulent phages to acquire genes from temperate phages, which often carry virulence genes, is reassuring for the development of phage therapy as a means to combat bacterial infections. It should be noted however that some virulent phages are in fact former temperate phages that lost their lysogeny module. The case is well documented among dairy phages [75]. Such “virulent” phages, that should rather be named “ex-temperate”, do exchange sequences with temperate and defective prophages [75] and should therefore be avoided for phage therapy, as already indicated [76]. To ascertain the choice of virulent phages for therapeutic use in the future, it may be relevant to conduct an analysis similar to the one presented here, by looking for mosaics (*i.e.* Blast matches above 90% identity) between the selected (and sequenced) phage, and all possible sequenced phages and prophages infecting the targeted bacterial species.

### Consequences for evolution

The relaxed exchange of genetic information among phages might result from different selective pressures acting on viruses as compared to bacteria. The level of HR is indeed the result of contradictory demands. Recombination between homologous sequences must be an efficient process to repair rapidly DNA lesions. On the other hand, recombination between different homologous loci, by creating genomic rearrangement, can destroy chromosomal integrity and functional gene associations and must be avoided. This problem is especially pronounced in eukaryotes, which contain repeated sequences with slight polymorphism, that are prone to recombine together, but bacterial chromosomes also possess repeated elements and related genes. Phage genomes, however, do not possess repeated sequences usually, and can thus better tolerate recombination between diverged sequences without risking chromosomal rearrangements. This might explain the phage tolerance to low fidelity recombination, which then accidentally contribute to rapid phage evolution. Alternatively, as recombination of diverged sequences is sometime advantageous for phages, it is tempting to speculate that it is under positive selection. First, high levels of shuffling inside genes can generate new functions (reviewed in [77]). We found that small sequences of 100 bp within genes are frequently exchanged. This mechanism to generate new genes could explain the much larger viral gene repertoire as compared to bacterial gene repertoire. Secondly, creation of new gene combinations is likely valuable in numerous functions to allow greater phage propagation, e.g., escape from CRISPR-mediated bacterial immunity systems [78] or expansion of bacterial host range [72], [79].

Temperate phage genes are alternatively submitted to very different selective pressures depending on the nature of their replicating cycle. Once integrated, prophage DNA becomes subject to the selective forces working on the bacterial chromosome. This explains that temperate phages constitute a reservoir of genes that improve bacterial phenotypes by lysogenisation [76], [80]. Likewise, defective prophages, that were long considered to be mere genetic junk, are now known to confer a broad range of beneficial phenotypes to the bacterial hosts, with respect to virulence, stress resistance, or even mutation rate [80]–[83]. The present study illustrates that inversely, prophage remnants can be a reservoir of functional lytic cycle genes for temperate phages. Dlp12 lysis module, helpful in certain strains of *E. coli* for biofilm development [43], is also active in λ for lysis. The constant exchange of genes between prophage remnants entrapped in host chromosome and active phages, *via* homologous recombination, blurs even more the distinction between evolutionary pressures acting on temperate phages and their bacterial host, tightly links their evolution, and indirectly accelerates bacterial evolution itself.

## Materials and Methods

### Bacterial and phage strains

All phage and bacterial strains are listed in Table S1. Unless specified, all gene replacements, deletions or insertions were done by recombineering as already described [84], with primers specified in Table S2.

The MG1655 *stfR*::c*at* (in Rac prophage) and *tfaQ*::*cat* (in Qin prophage) were constructed by inserting the *cat* cassette from the pKD3 plasmid into the respective genes with the *stfR::cat* and *tfaQ::cat* oligonucleotide pairs listed in Table S2, respectively. The MG1655 *ybcV*::*Kan* (in Dlp12 prophage) was constructed by P1 transduction from the Keio collection strain ECK0550 [85].

The Urλ*ble* phage was constructed from Urλ isolated from the *E. coli* K12 ancestral strain. The phleomycine/bleomycine resistance gene *ble*, cloned under the *Bacillus subtilis* promoter p*sacB*, taken from plasmid pUCphleo (gift from E. Dervyn), was introduced between the *tfa* and *ea47* genes of λ, and oriented as *tfa*. The presence of *ble* does not modify phage growth (Fig. S2), nor the frequency of lysogenization. Complete sequencing of Urλ*ble* showed that no important mutation other than the presence of the *ble* gene and the expected frameshift in the *stf* gene [40] differentiate our strain from the sequenced λPaPa strain (Table S2).

Deletions of *redβ*, *redα*, *orf* and *rap* genes were done by recombineering in *E. coli* K12 Urλ*ble*. In the case of the deletion of *redβ*, a RBS was introduced to maintain the expression of *redα*, as the two genes are overlapping and *redα* RBS is inside *redβ*.

Phage Φ80 was isolated in our laboratory from a strain contamination. Phage Φ80*ble* was constructed by introducing the p*sacB*-*ble* construct after *gp63*, and in the same orientation.

The λNec9 and 10 were constructed by replacing *redα* and *redβ* by the Rac-encoded *recE* and *recT* genes, in λNec4 and λNec6, respectively (phage strains listed in Table S1). To do this, the fragment corresponding to the C-terminal (from a.a. 588) of RecE and the full *recT* gene was added into pKD4 at the *Bmg*BI site, to give pJA100. The PCR fragment generated from pJA100, using the pair of oligonucleotides pKD4 (Table S3) was then introduced between the *gam* and *orf60a* genes of λNec4 and λNec6 by recombineering [84]. To construct λNec11 and λNec12, *redα* and *redβ* were replaced by *erf* and *exo* genes from D3 (a *P. aeruginosa* phage) in λNec4 and λNec6, respectively. First, these two genes were cloned into pKD4 (pJA82, constructed with erf/exo oligonucleotides described in Table S3) at the *Bmg*BI site, then the PCR fragment generated from this plasmid (using the oligonucleotides pKD4 described in Table S3) was introduced between the *gam* and *orf60a* genes of λNec4 and λNec6, following the same steps as for the *recET* constructions. Final constructs were verified by sequencing.

### Measure of phage growth

Burst size was determined on *E. coli* MG1655 bacteria growing exponentially in LB broth supplemented with maltose (0.2% w/v) and magnesium (MgSO4 at a concentration of 10 mM). When OD_600_ reached 0.2, 10 ml of the culture were concentrated 10 times, and the phage added at a multiplicity of infection of 0.002. The mix was incubated for 7 minutes at 37°C and then diluted 100-fold and 10,000-fold in LB at 37°C. The number of non-adsorbed phage at the start of the phage growth was evaluated by plaque assay after bacterial centrifugation. Samples of the two dilution mixes were taken repeatedly throughout time and assayed immediately for plaque-forming units. The burst size is the factor between final phage number and initial phage number, subtracting the number of non-adsorbed phage at time of dilution.

Plaque size was determined after overnight growth on a layer of *E. coli* MG1655 bacteria embedded into top agarose supplemented with maltose and magnesium (2 g/L agarose, 10 g/L tryptone, 5 g/L yeast extract, 5 g/L NaCl, 10 mM MgSO4, 2 g/L maltose). Plates imaging and analysis was realized with the Colony Doc-it imaging station (UVP, Upland, Canada) with the same settings for all plates.

### Phage-prophage recombination assay

The phage to be tested was multiplied on the appropriate *E. coli* strains on plates. Briefly, 10^4^ (for Red+ λ strains and Φ80 strains) or 10^6^ (for Δ*redβ* λ strains) PFU were mixed to 100 µl of overnight bacterial culture grown on LB maltose. After 5 minutes of incubation, 4 ml of top agar containing MgSO4 10 mM were added and then poured on a fresh LBA plate containing 0.2% of glucose. After 6 to 8 hours of incubation at 37°C, when lysis was confluent, 5 ml of water were poured on the top of the plates and incubated at 4°C for two hours. The phage supernatant was then recovered and filtrated at 0.2 µm. Stocks were around 10^10^ PFU/ml for Red positive strains and 10^9^ PFU/ml for Δ*redβ* or Δ*exo* strains. A theoretical calculation based on the measured burst sizes in liquid medium allowed to estimate that under such conditions Redβ^+^ and Redβ^−^ phages performed similarly 2.8 generations during the recombination assay. Φ80 experiments were performed similarly but with few differences: no maltose was added to the medium, and the same amount of RecT+ and RecT− phages (10^4^) were used for inoculation as no difference in plaque sizes was observed between the two genotypes.

### Read-out of the phage-prophage recombination assay by lysogenization

1 ml of *E. coli* MG1655 culture growing in LB+0.2% maltose+ MgSO4 10 mM was added to the phage stock in which recombinants were produced (m.o.i = 1) when the OD_600_ of the culture reached 1. The mix was then incubated for 1.5 hours at 37°C in a closed 2 ml tube. The total number of lysogenized bacteria was measured by plating the bacteria at the appropriate dilution on phleomycine (5 mg/L) plates. Recombinant phage concentration was estimated by plating on either chloramphenicol (20 mg/L) or kanamycine (50 ml/L) plates. In these conditions, 5 to 10% of the bacteria were lysogenized, as indicated by the proportion of bacteria that acquired phleomycine resistance. Recombinant frequencies are the ratio of chloramphenicol or kanamycine resistant bacteria on phleomycine resistant bacteria. Figures given are an average of at least three independent recombination assays, followed for each of them by at least 3 read outs by lysogenisation.

### Analyses of recombinant clones

PCR amplification of the λ genomic region susceptible of containing the resistance gene acquired from the host chromosome, if recombination was guided by homology, were realized on more than 20 colonies per genotype (oligonucleotide sequences are given in Table S3). For each colony tested, a fragment at the expected size was obtained. About 35% of the colonies gave an additional band corresponding to the native size of the λ region tested. A PCR using divergent oligonucleotides at *int* and *ea59* (that give a product if λ is excised or integrated in multiple copies) allowed confirming that these colonies were polylysogens. As the same proportion of polylysogens was found for each mutant, this phenomenon was neglected for the calculation of the frequency of recombinants.

### *P_L_* inversion assay on phage λ

2×10^6^ λNec phages (100 µL) were incubated 5 min with 2×10^7^ (500 µL) of exponentially growing C600 *recA* cells. Then 5 mL of top agar containing 10 mM MgSO4 was poured onto the mix and plated on LB plates, which were incubated 6 h at 37°C, until confluent lysis. 5 mL of a 10 mM MgSO4 solution were poured on the lysed plates, and the whole top agar layer was recovered, vortexed, and centrifuged for 10 min at 4°C. The supernatant, containing the phage, was then recovered and filtered at 0.2 µm. To estimate the amount of recombinants produced during lytic growth on plates, the stock was titrated on C600 *recA* strain to count parental phage, and on a C600 P2 lysogen strain, where only recombinant phage can grow, to count the recombinants. The frequency of recombination was then calculated by the number of recombinant PFU divided by the number of PFU corresponding to parental phages, counted on the *recA* strain.

### Bioinformatics analysis of mosaicism

A set of 50 non-redundant genomes (less than 92% overall identity or coverage <80%) of phages infecting *E. coli*, and having either temperate (n = 24) or virulent (n = 26) lifestyles were chosen (see Table S4). The 34 non-redundant defective prophages were those of strains MG1655 and 0157:H7 Sakai [37] as well as the prophages identified in IAI39 *E. coli* strain (all contained Insertion Sequences (IS) in genes important for the phage life cycle, and were therefore classified as defective). The analysis was focused on recent exchanges only (mosaics with more than 90% identity), because it aimed at detecting traces of recombination in mosaic flanking sequences. Among the selected phages, some pairs were too closely related to contribute to the analysis: when more than 50% of the smaller genome of the pair shared more than 70% average nucleotide identity with its partner, the pair was excluded (black squares in Fig. 6, the strategy used to examine phage relatedness is shown Fig. S7). To detect recent exchanges, a Blastn was run on each genome pair, and all hits of a minimal size of 100 bp and sharing more than 90% identity were selected. Among these hits, some corresponded to IS sequences: they contributed for 9% of the total hits found among temperate phages, but as much as 33% among the defective phage pairs. The rest of the hits were named mosaics.

Under the hypothesis of mosaics formed by homologous recombination between diverged sequences, the scenario depicted in Fig. 7 is supposed to take place. Two ancestral phages A and B sharing in average 60% identity except for some more conserved regions at 80% identity, recombine across these 80% identical sequences (light grey). As a consequence, the new piece of DNA in the recombined phage C, when compared to its parent B providing the mosaic, exhibits a 100% identity region (dark grey), flanked by the two 80% identical sequences, above the background level of 60% identity shared by the two genomes at the time of exchange, and somewhat less at the time where genomes are analyzed. Following this scheme, to detect traces of homologous recombination at the vicinity of the mosaics, pairs of 2 kb-long sequences flanking the mosaics were realigned by dynamic programming, and the identity level of successive 50 bp-long windows along the alignment was measured. The 50 bp length was chosen as a close value to the minimum required for Redβ recombination (30 bp). The probability to encounter such HR traces at random (last line of Table 1) was calculated as described in Text S1.

## Supporting Information

Figure S1 Distribution of perfect identity segment sizes in the different homology regions. Lengths of homology regions with λ (A) and Φ80 (B) are shown in blue and red for the regions placed left and right from the antibiotic cassette, respectively. Green bars indicate the segments in which a recombination event was detected. (C) Purple: Length distribution of homology regions for the *oxa5/oxa7* sequences (P*_L_* inversion assay). Green: segments in which a recombination event was detected. (D) Numbers of MEPS (Minimal Efficiency Pairing Segment), defined as the minimal length of strict identity to initiate pairing, for Redβ and RecA in the different homology regions.
(DOCX)

Figure S2 Single burst growths of Urλ, Urλ*ble* and Urλ*ble* Δ*redβ* (λSOC27). The insertion of the phleomycin resistance gene (*ble*) does not modify either the burst size or the latency period of the phage. Deletion of the *redβ* gene reduces the burst size by 5 fold, but the latency period is unchanged. The experiment has been repeated four times independently, and a representative result is shown.
(DOCX)

Figure S3 Examination of Rac prophage replication. As the Rac prophage possesses an active origin of replication, *oriJ*, we investigated by semi-quantitative PCR if λ infection activates Rac replication. Cells infected or not with λ at an m.o.i. of 1, were harvested 30 min after infection for total DNA purification. DNA was purified after phenol-chloroform extraction and ethanol precipitation steps. PCR was realized on 50 pg of total DNA, with 22 cycles of amplification. 1 to 2: PCR with primers framing *oriJ* does not indicate Rac replication following λ infection (line 2, MG+λ) compared to the non-infected MG1655 strain (line 1, MG). 3 to 4: internal control with primers within the *gyrB* gene. 5 & 6: PCR on the λ gene Q shows λ replication in the MG+ λ sample (lane 6).
(DOCX)

Figure S4 Plaque size distribution of λ mutants in the presence or absence of RecA in the host. To determine the effect of phage mutations on growth, we used the plaque size method. As all phage parameters like virion size, adsorption rate and latency period are equal in other respects, a reduction in burst size should directly translate into a smaller plaque of lysis on a lawn of bacteria. The same phage mutants were thus grown on MG1655 or isogenic *recA* lawns. *redβ and redα* mutants give a 2.5-fold smaller plaque area, but no difference in plaque size for *orf* and *rap* mutants is observed. On a *recA* lawn, a uniform and modest 15% decrease of plaque size was found for all mutants, probably reflecting the lower growth rate of the *recA* mutant. Interestingly, on a *recA* host, the *redβ* deleted phage makes plaques of a similar size compared to the WT host, whereas homologous recombination on phage DNA is abolished. This suggests that RecA, while being essential for HR when a λ *red* phage is used, is dispensable for growth of such λ *red* phage, and therefore that the Red function in growth is distinct from its role in homologous recombination.
(DOCX)

Figure S5 Distribution of mosaic sizes in the 4 categories of comparisons.
(DOCX)

Figure S6 Strategy to determine the phage genome pairs to be excluded from the mosaic analysis. Mosaic analysis was focused on recent exchanges (mosaics with more than 90% identity), because it aimed at detecting the relatedness of the mosaic flanking sequences. The genome pairs too closely related to permit detection of such flanking sequences needed to be eliminated. We reasoned that if the average nucleotide identity (ANI) of the genome pair was above 70%, no clear signal above the ANI value, and different from the mosaic signal would be detected. We also specified that at least 50% of the genomes needed to be included for the measurement of the ANI value, to be relevant. To measure the ANI of each genome pair, the method of Goris et al. was adapted [86]. Instead of using standard Blast parameters, with which ANI below 80% are seldom generated, we used the following values: cost to open a gap: 5, cost to extend a gap: 2, penalty for nucleotide mismatch : - 4, reward for nucleotide match : 3, word size : 10. This allowed reaching ANI values down to 60%. ANI values as a function of genome coverage for temperate and defective pairs (**A**), and for virulent pairs (**B**) are shown. The virulent pairs of genomes are dominated by T4-like phages, which group nicely into discrete sets of ANI values. In order to avoid cutting within a group, virulent pairs with ANI value above 68% and coverage above 48% were removed from the mosaic analysis (red frame). In a marked contrast, temperate/defective pairs form a cloud of points, among which the subset with ANI value above 70% and coverage value above 50% are the pairs removed from the analysis (red frame). ANI analysis was performed for pairs of genomes listed in TableS4 having mosaics. In the black frames of Figure S6, (>92% id, >80% cov), genomes are so similar that they were completely removed from the analysis (indicated with * as redundant in the last column of Table S4).
(DOCX)

Figure S7 Scheme for the masking of homologous recombination traces. Genome A/B is the product of a recombination between A and B in regions of homology. As described in Fig. 7, alignment of the two genomes should reveal traces of homologous recombination on both sides of the mosaic. However, recombination of the A/B hybrid with a third genome, C, can mask one of the two HR traces. Alignment of A/B/C hybrid with the ancestral B genome will reveal only one trace of homologous recombination.
(DOCX)

Table S1 Bacterial and phage strains used in this study.
(DOCX)

Table S2 Differences between the sequence of λ (λPapa) and the Urλ-*ble* phage used in this study.
(DOCX)

Table S3 Primers used in this study.
(DOCX)

Table S4 List of phage genomes used.
(DOCX)

Text S1 Probability to encounter traces of HR at random around mosaics.
(DOCX)
