# Supplementary figures and images for: Temperate Phages Acquire DNA from Defective Prophages by Relaxed Homologous Recombination: The Role of Rad52-Like Recombinases
Source: PLoS Genet. 2014 Mar 6;10(3):e1004181. doi: 10.1371/journal.pgen.1004181 (PMC3945230; doi:10.1371/journal.pgen.1004181)

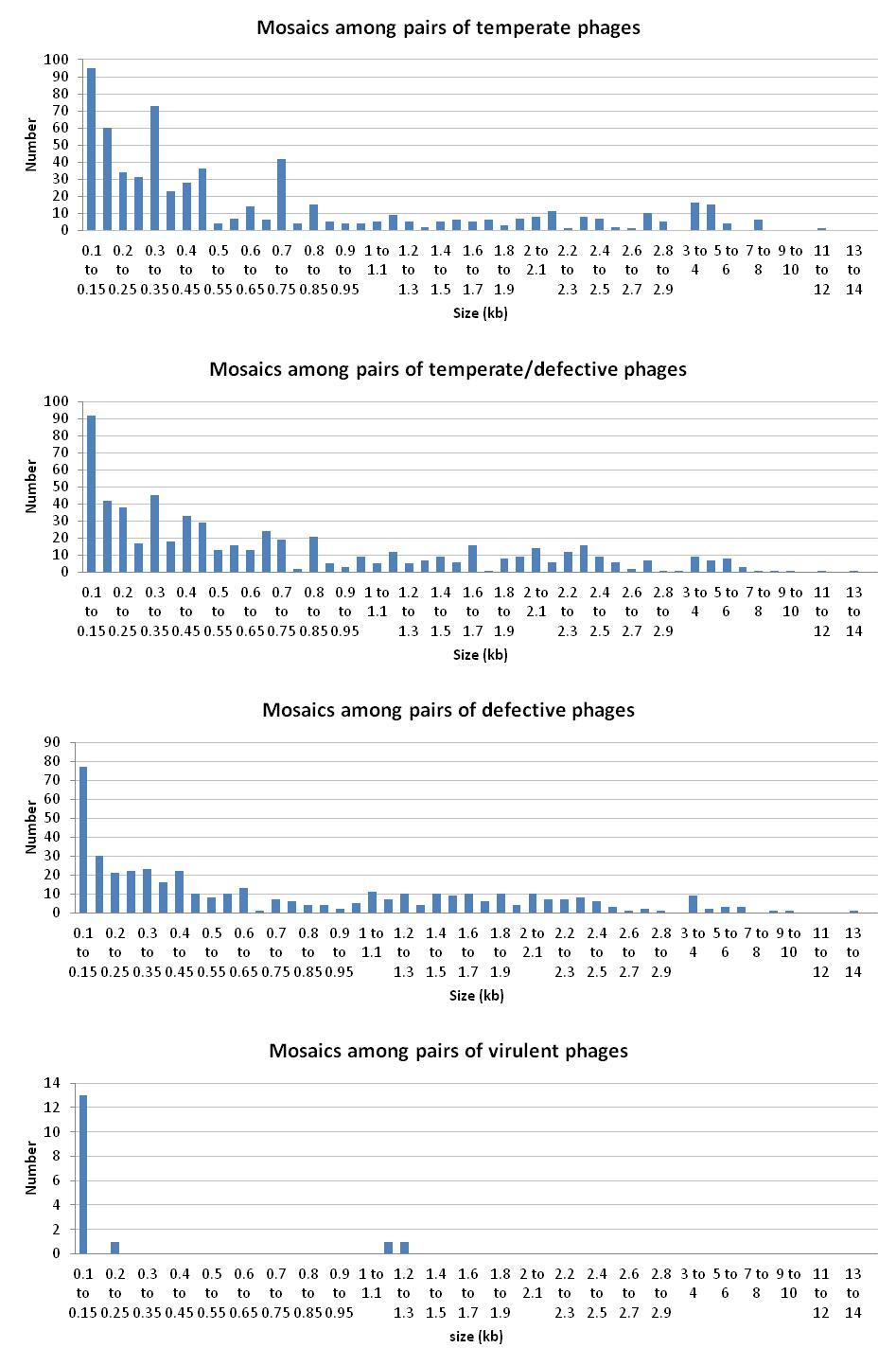


Figure S5

Supplement: Figure S5 — Distribution of mosaic sizes in the 4 categories of comparisons. (DOCX) [file pgen.1004181.s005.docx]
