# Supplementary material for: Temperate Phages Acquire DNA from Defective Prophages by Relaxed Homologous Recombination: The Role of Rad52-Like Recombinases
Source: PLoS Genet. 2014 Mar 6;10(3):e1004181. doi: 10.1371/journal.pgen.1004181 (PMC3945230; doi:10.1371/journal.pgen.1004181)
Supplement: Table S1 — Bacterial and phage strains used in this study. (DOCX) [file pgen.1004181.s008.docx]

**Table S1**: bacterial and phage strains used in this study.

| Strain | Genotype | Commentary |
| --- | --- | --- |
| MAC1117 | MG1655 *ilvD:cat* | gift from S. Delmas (INSERM U1001, Paris, France) |
| MAC1400 | MG1655 *stfR::cat* | this study |
| MD7 | MG1655 *stfR::cat* recA306 *srl::Tn10* | this study |
| MD19 | MG1655 *tfaQ::cat* | this study |
| MD63 | MG1655 *tfaQ::cat recA306 srl::Tn10* | this study |
| MD36 | MG1655 *ybcV::Kan* | this study. Allele from Keio collection |
| MD61 | ybcV::Kan *recA306 srl::Tn10* | this study |
| MD22 | MG1655 *yecD::cat* | this study |
| MD58 | MG1655 *nohD::cat* | this study |
| MD68 | MG1655 *ybcN::cat* | this study |
| MAC | C600 P2 lysogen | [19] |
| MAC | C600 recA306 *srl::Tn10* | [19] |
| Phage strains | | |
| λMD 50 | λPaPa | reference laboratory strain of phage λ . Gift from D. Refardt ([Refardt & Rainey, 2010](#_ENREF_2))^e^ |
| λMD51 | λPaPa *ble* | this study |
| Urλ | Urλ | Original isolates of λ. From the *E. coli* K12 strain of the *E. Coli* Genetic Stock Center. |
| Urλ *ble* | Urλ *ble* | Urλ strain carrying the *ble* gene that confers resistance to phleomycine. gift from L. Le Chat |
| λSOC 27 | Urλ *ble* *redβ::FRT* | this study |
| λSOC 28 | Urλ *ble* *exo::FRT* | this study |
| λSOC 29 | Urλ *ble orf::FRT* | this study |
| λSOC 30 | Urλ *ble rap::FRT* | this study |
| Φ80 MD2 | Φ80 *ble* | this study |
| λNec4 | [800bp IR, 100% id]^a^ cI857ts bΔ(22297-27728) | [19] |
| λNec1 | [invPL, 800bp IR, 100% id]^b^cI857ts bΔ(22297-27728) | [19] |
| λNec6 | [800 bp IR, 78%id]^c^ cI857ts bΔ(22050-26766) | [19] |
| λNec3 | [invPL, 800 bp IR, 78%id]^d^ cI857ts Δ(22050-26766) | [19] |
| λNec9 | [800bp IR, 100% id] cI857ts Δ(22297-27728) Δ*red::recET* | this study |
| λNec10 | [800 bp IR, 78%id] cI857ts Δ(22050-26766) Δ*red::* *recET* | this study |
| λNec11 | [800bp IR, 100% id] cI857ts Δ(22297-27728) Δ*red*::*erf-exo* | this study |
| λNec12 | [800 bp IR, 78%id] cI857ts Δ(22050-26766) Δ*red::* *erf-exo* | this study |

a. [800bp IR, 100% id] : recombination cassette composed of 800 bp-long inverted and 100% identical repeats flanking the pL promoter in its native orientation. The genotype of this region is ea10:oxa7 rexA:(oxa7-cat-χ+) Δorf28-ral

b. [invPL, 800bp IR, 100% id]: same as a., but inverted orientation of the sequences inside the IR, including PL.

c. [800bp IR, 78% id] : recombination cassette composed of 800 bp-long inverted and 78% identical repeats flanking the pL promoter in its native orientation. The genotype of this region is ea10:oxa7-5 rexA:(oxa5-7-cat-χ+) Δorf28-ral

d. [invPL, 800bp IR, 78% id] : same as c, but inverted orientation of the sequences inside the IR, including PL. The genotype of this region is ea10:oxa7 rexA:(oxa5-cat-χ+) Δorf28-ral

e. Refardt D, Rainey PB (2010) Tuning a genetic switch: experimental evolution and natural variation of prophage induction. *Evolution* **64:** 1086-1097
