# Supplementary material for: Temperate Phages Acquire DNA from Defective Prophages by Relaxed Homologous Recombination: The Role of Rad52-Like Recombinases
Source: PLoS Genet. 2014 Mar 6;10(3):e1004181. doi: 10.1371/journal.pgen.1004181 (PMC3945230; doi:10.1371/journal.pgen.1004181)
Supplement: Table S2 — Differences between the sequence of λ (λPapa) and the Urλ-ble phage used in this study. (DOCX) [file pgen.1004181.s009.docx]

**Table S2**: Differences between the published sequence of λ and the Urλ-*ble* phage used in this study.

| λPaPa  NC_001416 | Ur-λ*ble* | orf | Change* |
| --- | --- | --- | --- |
| 138 | - G | IR *cos-nu1* | - 1 |
| 542 | C -> T | *nu1* |  |
| after 14267 | + G | IR *L-K* | + 1 |
| 17183 | A -> G | *J* | E_560_ -> G |
| 18835 | T -> G | *J* | Y_1111_-> D |
| 20661 | A -> G | *stf* |  |
| after 20835 | + C | *stf* | + 1 |
| 21714 | G -> A | *stf* | S_689_ -> N |
| 22445 | A -> G | *tfa* | K_158_ -> E |
| *22597-22633* | ***deletion -> psacB-phleoR*** | | |
| 25662 | T -> G | *ea59* | M_438_ -> L |
| 31016 | T -> C | *orf61* |  |
| 34934 | A -> G | *sieB* |  |
| 45618 | T -> C | *R* |  |

IR: intergenic region

*: Absence of change indicates a synonymous mutation. In addition to the expected C addition in *stf*, that restores the reading frame and therefore intact side tail fibers in Urλ, one deletion and two insertions are observed in intergenic regions (IR). Among the 8 substitutions, 3 are neutral, and 4 of the 5 non-synonymous changes are in tail component genes.
