# Supplementary material for: Temperate Phages Acquire DNA from Defective Prophages by Relaxed Homologous Recombination: The Role of Rad52-Like Recombinases
Source: PLoS Genet. 2014 Mar 6;10(3):e1004181. doi: 10.1371/journal.pgen.1004181 (PMC3945230; doi:10.1371/journal.pgen.1004181)
Supplement: Table S3 — Primers used in this study. (DOCX) [file pgen.1004181.s010.docx]

**Table S3: primers used in this study^. a^**

| Gene deletions or insertions | | |
| --- | --- | --- |
| stfR::cat | CGCCGTCACATGCCGGGACCATTACCGTGTATGAAGATTCCAACTTTTGGCGAAAATGAG | |
|  | GTTGATGCCGTTCCTGCGCTGGAAGACGCTGACTGAGCCGACGGGAACTTCATTTAAATGGCG | |
| tfaQ::cat | GTTATTTATTTCTGAACTCGGTCCGTTACCGGAAAATGTTACCTGTCCTCCTTAGTTCCTATTCC | |
|  | TATCCTTCACCCAGGCTGTGCCGTTCCACTTCTGAAACTCCCCTTCTGCCACTCATCGCAGTAC | |
| redβ::FRT | GATCCCGGTACGCTGCAGGATAATGTCCGGTGTCATGCTGTCCTCCTTAGTTCCTATTCC | |
|  | CATTGCTCACCACCAGGTTGATATTGATTCAGAGGTATAAAACGAGTGTAGGCTGGAGCTGCTTC | |
| exo::FRT | CTTGGATTCCTGAAACAGAAAGCCGCAGAGCAGAAGGTGGCAGCATGAACACAGGAACACTTAACGGC | |
|  | CCTACCCGGATATTATCGTGAGGATGCGTCATCGCCATTGCTCCCCTGTAGGCTGGAGCTGCTTCG | |
| orf::FRT | GGGAGAGGGAAGTCATGAAAAAACTAACCTTTGAAATTCGGCAGCATTACACGTCTTGAG | |
|  | CATGCAGCCCTGTCTCCCCATCTCGCTTTCCACTCCAGAGCCCATGGTCCATATGAATATCCTCC | |
| rap::FRT | GATGATGGCTAAACCAGCGCGAAGACGATGTAAAAACGATGGCAGCATTACACGTCTTGAG | |
|  | GTCATGCGGCCTCACTTCTGCTATTTCGCAGGTCTTTGAGCCATGGTCCATATGAATATCCTCC | |
| Φ80-*ble* | CAACAAAGAGATAAATCCAAGCCCGTTTTGTACGGGCTGTatgaccatgattacgccaag | |
|  | ATGCAGGTAACGCAGCCAGAATTGTTGATTGGCTGATCGTTAAGTTGGGTAACGCCAGG | |
| yecD::cat | CTTCCACGCTACGCACACGGGCGATGCGCGGGTAGATATGGTTGACAACTTTTGGCGAAAATGAG | |
|  | AAGACGCCTGTAGTGCCGCCAGCGCCGAGCAGCACAATAACAGTAGGAACTTCATTTAAATGGCG | |
| nohD::cat | ATTGAGTACGAACGCCATCGACTTACGCGTGCGCAGGCCGATGCACAGGACAACTTTTGGCGAAAATGAG | |
|  | CACCTGCGATCCGCGACAGCACGAAAGTACAGAATGCGGTTTCCACCACTGGAACTTCATTTAAATGGCG | |
| ybcN::cat | CTCCAGCTTGGGTAAAAGATGCTCTCAAACACACATATCTCGGTTATGAAGGAACTTCATTTAAATGGCG | |
|  | TCGGAGGTATGGCGTAACGACTGGATAGTGGTGATATCACCGGTTACGACACAACTTTTGGCGAAAATGAG | |
| Detection of excision of defective prophages | | |
| Rac | CTCCAGCATGGTATAGCTGTCTTTAC | described in [82] |
|  | CAGATTTCTTATGCTGGGCGTTCCG |  |
| Qin | CGACAATACGCGCCACATAA |  |
|  | AACGGCGAGTAAGTAGTACGC |  |
| Dlp12 | CAAAAGCCATTGACTCAGCAAGG |  |
|  | ACGGATAAGACGGGCATAAATGA |  |
| Activity of oriJ (rac), semi-qPCR | | |
| *gyrB* | GTCGAAGTGGCGTTGCAGTG | described in ([Fogg et al, 2010](#_ENREF_1)) ^b^ |
|  | AGCCTGCCAGGTGAGTACCG |  |
| λ *Q* | GAGTGCGGAAGATGCAAAGG |  |
|  | TTAACAGTGCGTGACCAGG |  |
| *oriJ* | GTTGAGTCAGGCGAAATCC | |
|  | CCACGGTAACCACAATCAC |  |
| Identification of recombination junctions in λ hybrids | | |
| Qin | GCTCATGCCCATTCTTTC | |
|  | ACGTTTCTGCGGCATAT | |
| Rac | ACTGGCGTACTGACGGATTC (rac 1) | |
|  | ACGTTTCTGCGGCATAT (rac 2) | |
|  | ACGTTTCTGCGGCATATC (rac 3) | |
| Dlp12 | TTAGCATCCGCCCATTCAAC | |
|  | TGGTCAGTTCGAGCATAAGG | |
| Construction of *recET* and *exo/erf* λ | | |
| recE | CGAGCTCCCGCTCGAGCTTTTGCGATTGGTGGTTGC | |
|  | GGAGGATGAAACTGGCGAAGTCGCA | |
| recT | CGAGCTCCATTACACCGCCAGGCTGAA | |
|  | CCGCTCGAGGGAGGCTGGGCTAAGGAATATGCAA | |
| exo/erf | GTTTGCCGCATGTGGCTTTG | |
|  | GGAGGCCATCAACAACGAAGCCC TG | |
| pKD4 | TGATTGCGCCTACCCGGATATTATCGTGAGGATGCGTCATTCCTCCTTAGTTCCTATTCC | |
|  | CATTGCTCACCACCAGGTTGATATTGATTCAGAGGTATAATGCAGATTGCAGCAT TACAC | |

1. Unless specified, primers were designed for this study.
2. Fogg PC, Allison HE, Saunders JR, McCarthy AJ (2010). *J Virol* **84:** 6876-6879
