# Supplementary material for: Temperate Phages Acquire DNA from Defective Prophages by Relaxed Homologous Recombination: The Role of Rad52-Like Recombinases
Source: PLoS Genet. 2014 Mar 6;10(3):e1004181. doi: 10.1371/journal.pgen.1004181 (PMC3945230; doi:10.1371/journal.pgen.1004181)
Supplement: Table S4 — List of phage genomes used. (DOCX) [file pgen.1004181.s011.docx]

**Table S4: List of phage genomes used.**

| Phage | length (bp) | life style^a^ | family | accession number, *redundancy with |
| --- | --- | --- | --- | --- |
| 186 | 30624 | T | P2 | NC_001317 |
| 536 | 46023 | T | λ | NC_008253(1189882..1228488) |
| 86 | 60238 | T | λ | NC_008464 |
| 933W | 61670 | T | λ | NC_000924 , *VT2Sakai |
| BP-4795 | 57930 | T | λ | NC_004813 |
| Bp7 | 168066 | V | T4 | NC_019500 |
| CC31 | 165540 | V | T4 | NC_014662 |
| cdtI | 47021 | T | λ | NC_009514 |
| CP4-57 | 22030 | D | P2 | NC_000913.2(2753978..2776007) |
| CP4-6 | 34306 | D | fragments | NC_000913.2(262184..296489) |
| dlp12 | 21302 | D | λ | NC_000913.2(564025..585326) |
| e14 | 15193 | D | λ | NC_000913.2(1195443..1210646) |
| HK022 | 40751 | T | λ | NC_002166 |
| HK446 | 39026 | T | λ | NC_019714 |
| HK620 | 38297 | T | λ | NC_002730 |
| HK97 | 39732 | T | λ | NC_002167 |
| HX01 | 169158 | V | T4 | NC_018855, *RB69 |
| IAI39-Ph01 | 32853 | D | λ | NC_011750.1(532595..565447) |
| IAI39-Ph02 | 12130 | D | λ | NC_011750.1(923080..935209) |
| IAI39-Ph03 | 28321 | D | λ | NC_011750.1(1555796..1584116) |
| IAI39-Ph04 | 18544 | D | P2 | NC_011750.1(1882552..1901095) |
| IAI39-Ph05 | 23629 | D | λ | NC_011750.1(1932603..1956231) |
| IAI39-Ph06 | 55581 | D | unknown | NC_011750.1(1992456..2048036) |
| IAI39-Ph07 | 13835 | D | λ | NC_011750.1(2060694..2074528) |
| IAI39-Ph08 | 41562 | D | ε15 | NC_011750.1(2751408..2792969) |
| IAI39-Ph09 | 11389 | D | unknown | NC_011750.1(2934628..2946016) |
| IAI39-Ph10 | 14803 | D | Mu | NC_011750.1(4625205..4640007) |
| IAI39-Ph11 | 44948 | D | λ | NC_011750.1(5025091..5097038) |
| IME08 | 172253 | V | T4 | NC_014260 |
| jk06 | 46072 | V | T1 | NC_007291 |
| JS10 | 171451 | V | T4 | NC_012741, *JS98 |
| JS98 | 170523 | V | T4 | [NC_010105](http://www.ncbi.nlm.nih.gov/nuccore/161622381) |
| JSE | 166418 | V | T4 | [NC_012740](http://www.ncbi.nlm.nih.gov/nuccore/238694878), *RB49 |
| K1-5 | 44385 | V | Sp6 | NC_008152 |
| K1E | 45251 | V | Sp6 | NC_007637 |
| K1F | 39704 | V | T7 | [NC_007456](http://www.ncbi.nlm.nih.gov/nuccore/77118167) |
| KP15 | 174436 | V | T4 | [NC_014036](http://www.ncbi.nlm.nih.gov/nuccore/294661423) |
| KplE1 | 9687 | D | fragments | NC_000913.2(2464404..2474619) |
| lambda | 48502 | T | λ | NC_001416 |
| Mu | 36717 | T | Mu | NC_000929 |
| N15 | 46375 | T | N15 | NC_001901 |
| N4 | 70153 | V | N4 | NC_008720 |
| NJ01 | 77448 | V | C3 | NC_018835 |
| P1 | 94800 | T | P1 | NC_005856 |
| P2 | 33593 | T | P2 | NC_001895 |
| P22 | 41724 | T | P22 | NC_002371 |
| P27 | 42575 | T | unknown | NC_003356 |
| P4 | 11624 | T | P2 | NC_001609 |
| Phi1 | 164270 | V | T4 | [NC_009821](http://www.ncbi.nlm.nih.gov/nuccore/157311299) |
| phi80 | 46150 | T | λ | JX871397 |
| phiV10 | 39104 | T | ε15 | [NC_007804](http://www.ncbi.nlm.nih.gov/nuccore/155370093) |
| Qin | 20458 | D | λ | NC_000913.2(1630450..1646830) |
| rac | 23060 | D | λ | NC_000913.2(1409966..1433025) |
| RB14 | 165429 | V | T4 | [NC_012638](http://www.ncbi.nlm.nih.gov/nuccore/228861315), *T4 |
| RB15 | 166877 | V | T4 | phage.ggc.edu/gbrowse/RB15/RB15.fa, *T4 |
| RB16 | 176788 | V | T4 | [NC_014467](http://www.ncbi.nlm.nih.gov/nuccore/304373555) |
| RB18 | 166677 | V | T4 | phage.ggc.edu/gbrowse/RB18/RB18.fa, *T4 |
| RB26 | 163036 | V | T4 | phage.ggc.edu/gbrowse/RB26/RB26.fa, *T4 |
| RB32 | 165890 | V | T4 | [NC_008515](http://www.ncbi.nlm.nih.gov/nuccore/116326222), *T4 |
| RB43 | 180500 | V | T4 | [NC_007023](http://www.ncbi.nlm.nih.gov/nuccore/66391451) |
| RB49 | 164018 | V | T4 | [NC_005066](http://www.ncbi.nlm.nih.gov/nuccore/33620426) |
| RB51 | 168394 | V | T4 | [NC_012635](http://www.ncbi.nlm.nih.gov/nuccore/228860932), *T4 |
| RB69 | 167560 | V | T4 | [NC_004928](http://www.ncbi.nlm.nih.gov/nuccore/32453484) |
| RTP | 46219 | V | T1 | NC_007603 |
| rv5 | 137947 | V | RV5 | NC_011041 |
| sp1 | 10586 | D | unknown | NC_002695.1(300041..310626) |
| sp10 | 51112 | D | λ | NC_002695.1(1921414..1972525) |
| sp11 | 45778 | D | λ | NC_002695.1(2158174..2203951) |
| sp12 | 46142 | D | λ | NC_002695.1(2203952..2250093) |
| sp13 | 21120 | D | P2 | NC_002695.1(2592901..2614020) |
| sp14 | 44029 | D | λ | NC_002695.1(2668007..2712035) |
| sp15 | 47879 | T | λ | NC_002695.1(2895926..2943804) |
| sp16 | 8551 | D | unknown | NC_002695.1(3192983..3201533) |
| sp17 | 24199 | D | unknown | NC_002695.1(3475965..3500163) |
| sp18 | 38759 | D | Mu | NC_002695.1(5040843..5079601) |
| sp2 | 12887 | D | P2 | NC_002695.1(310627..323513) |
| sp3 | 38586 | D | λ | NC_002695.1(891123..929708) |
| sp4 | 49650 | D | λ | NC_002695.1(1161091..1210740) |
| sp5 | 62708 | T | λ | NC_002695.1(1246012..1308719), *VT2Sakai |
| sp6 | 48423 | D | λ | NC_002695.1(1541470..1589892) |
| sp7 | 15463 | D | unknown | NC_002695.1(1594570..1610032) |
| sp8 | 46897 | D | λ | NC_002695.1(1618153..1665049) |
| sp9 | 58175 | D | λ | NC_002695.1(1757506..1815680) |
| SPN3US | 240413 | V | unknown | JN641803 |
| stx2-phage1717 | 62147 | T | λ | NC_011357 |
| stx2-phageI | 61765 | T | λ | NC_003525, *VT2Sakai |
| T1 | 48836 | V | T1 | NC_005833 |
| T2 | 163833 | V | T4 | <http://phage.ggc.edu/gbrowse/T2/T2.fa>, *T4 |
| T3 | 38208 | V | T7 | NC_003298 |
| T4 | 168903 | V | T4 | NC_000866 |
| T4T | 168920 | V | T4 | http://phage.ggc.edu/gbrowse/T4T/T4T.fa |
| T5 | 121750 | V | T5 | NC_005859, *T4 |
| T6 | 168705 | V | T4 | http://phage.ggc.edu/gbrowse/T6/T6.fa, *T4 |
| T7 | 39937 | V | T7 | NC_001604 |
| TLS | 49902 | V | T1 | NC_009540 |
| vB-EcoM-ACG-C40 | 167396 | V | T4 | [NC_019399](http://www.ncbi.nlm.nih.gov/nuccore/414086362), *T4 |
| vB-EcoM-VR7 | 169285 | V | T4 | [NC_014792](http://www.ncbi.nlm.nih.gov/nuccore/314121562) |
| VT2-Sakai | 60942 | T | λ | NC_000902 |
| Wphi | 32684 | T | P2 | NC_005056 |
| wV7 | 166452 | V | T4 | [NC_019505](http://www.ncbi.nlm.nih.gov/nuccore/422934783), *T4 |
| YYZ-2008 | 54896 | T | λ | NC_011356 |

1. D, defective; T, temperate; V, virulent.
