# Supplementary material for: Temperate Phages Acquire DNA from Defective Prophages by Relaxed Homologous Recombination: The Role of Rad52-Like Recombinases
Source: PLoS Genet. 2014 Mar 6;10(3):e1004181. doi: 10.1371/journal.pgen.1004181 (PMC3945230; doi:10.1371/journal.pgen.1004181)
Supplement: Text S1 — Probability to encounter traces of HR at random around mosaics. (DOCX) [file pgen.1004181.s012.docx]

**Text S1: Probability to encounter traces of HR at random around mosaics :**


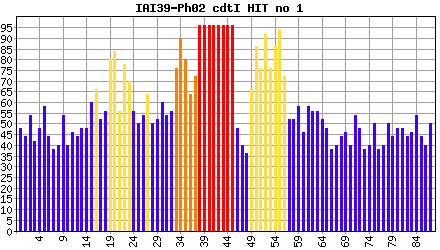
At the vicinity of the mosaics, pairs of 2 kb-long sequences flanking the mosaics were aligned, and the identity level of successive 50 bp-long windows along the alignment was measured. An example of the identity profile flanking a mosaic is shown below. In red, the mosaic, in orange, the flanking region that could be a trace of HR, and in yellow some other region of higher-than-background similarity, that does not flank a mosaic. In blue, the background level of identity of the two regions compared.

% identity

Window # in the alignment

From such identity profiles, ***b***, the background level of identity shared by the two genomes (taken from the third median value) is derived. Next, regions having a higher-than-background level of identity, namely above ***b***+10%, and placed next to the mosaic are classified as “trace of HR”, whereas the other such regions that are distant from the mosaic are classified as “bumps”. In the above example, the mosaic flanking region has 1 trace of HR and 4 bumps. For a given category of phages being compared, the total number of mosaics found is ***Nm***, the total number of traces of HR is ***Nth***, and the total number of bumps is ***Nb***. To estimate whether ***Nth*** is significantly above ***Nr***, the number of bumps expected at random just next to the mosaic, the following calculation is made: we model the occurrences of bumps along the concatenated realigned sequences by a Poisson process with intensity ***i*** estimated by the number of bumps ***Nb*** divided by the cumulative length of all sequences realigned. The number ***Nr*** of bumps expected at random in the 100 bp next to all mosaics of the group is then distributed according to a Poisson distribution with mean ***Nm*** x 100 x ***i***. It implies that the probability to observe as much as ***Nth*** traces of HR is given by the tail of this Poisson distribution and found to be in the 10^-14^- 10^-16^ range for all groups of phages tested (values reported in Table 1 of the main text). We conclude therefore that the number of traces of HR found next to the mosaics is highly significant. As a control, the same study was done on the hits corresponding to IS. 71 among 533 hits analysed contained a trace of homologous recombination at their boundary, for a random number expected ***Nr*** of 46. In this case, a p-value of 0.025, at the limit of significativity, was obtained, as expected for IS that should not exchange by HR but by transposition (unless degenerated).
