# Supplementary material for: Temperate Phages Acquire DNA from Defective Prophages by Relaxed Homologous Recombination: The Role of Rad52-Like Recombinases
Source: PLoS Genet. 2014 Mar 6;10(3):e1004181. doi: 10.1371/journal.pgen.1004181 (PMC3945230; doi:10.1371/journal.pgen.1004181)
Supplement: Supplementary file 8 [file pgen.1004181.s002.docx]

FIGURE S2

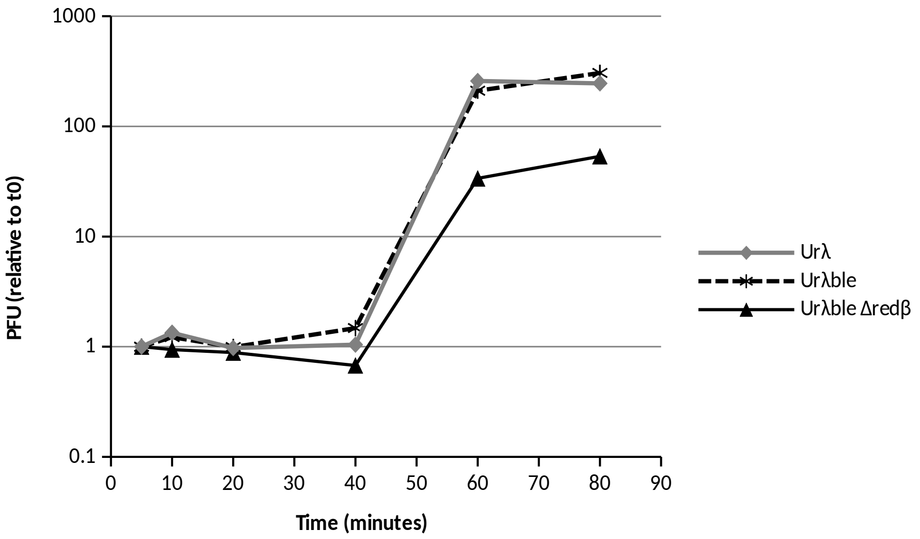

1000
100
10
1
0.1
0
10
20
30
40
50
60
70
80
90
PFU (relative to t0)
Time (minutes)
Urλ
Urλble
Urλble Δredβ
