# Supplementary material for: Temperate Phages Acquire DNA from Defective Prophages by Relaxed Homologous Recombination: The Role of Rad52-Like Recombinases
Source: PLoS Genet. 2014 Mar 6;10(3):e1004181. doi: 10.1371/journal.pgen.1004181 (PMC3945230; doi:10.1371/journal.pgen.1004181)
Supplement: Supplementary file 9 [file pgen.1004181.s003.docx]

FIGURE S3

1 2 3 4 5 6

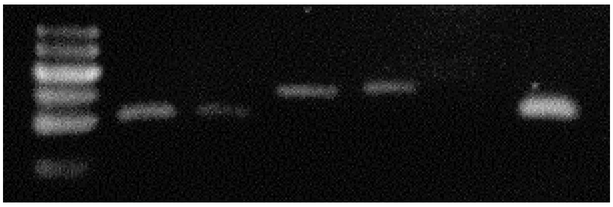

MG MG+λ MG MG+λ MG MG+λ

oriJ *gyrB* *λQ*

MG: MG1655 *E. coli* strain

MG+λ : MG1655 infected by λ
