# Supplementary material for: Temperate Phages Acquire DNA from Defective Prophages by Relaxed Homologous Recombination: The Role of Rad52-Like Recombinases
Source: PLoS Genet. 2014 Mar 6;10(3):e1004181. doi: 10.1371/journal.pgen.1004181 (PMC3945230; doi:10.1371/journal.pgen.1004181)
Supplement: Supplementary file 10 [file pgen.1004181.s004.docx]

FIGURE S4

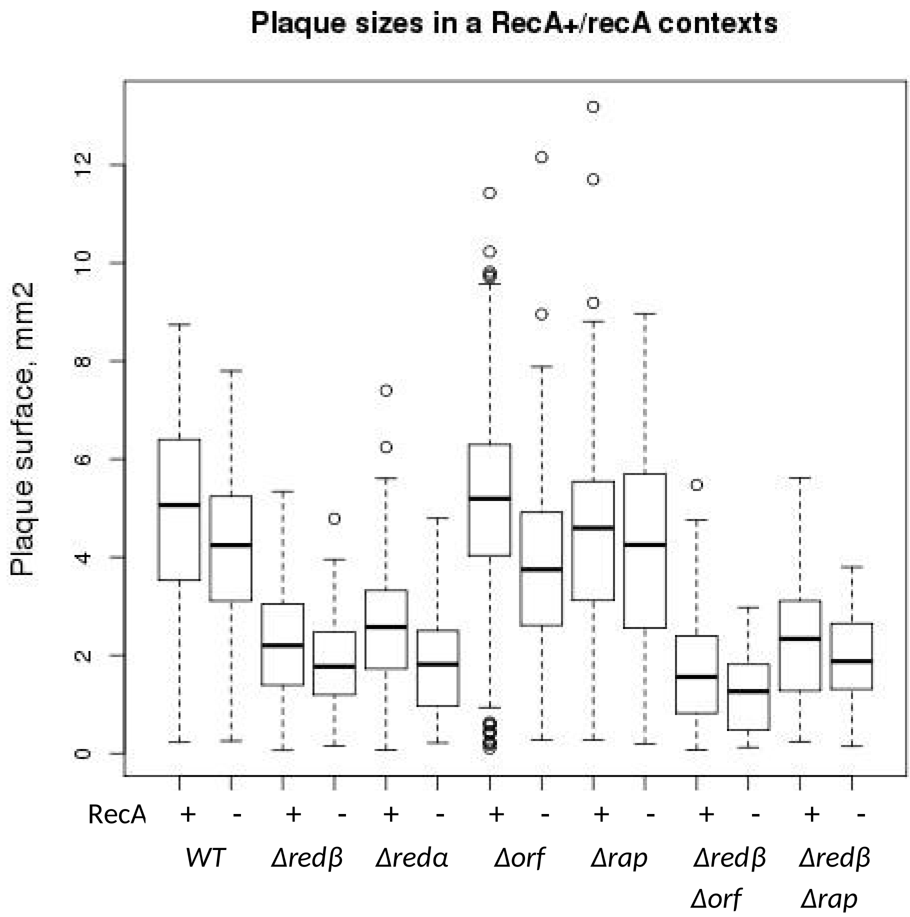

Plaque sizes in a RecA+/recA contexts
Plaque surface, mm2
0
2
4
6
8
10
12
RecA + - + - + - + - + - + - + -
WT
Δredβ
Δredα
Δorf
Δrap
Δredβ Δorf
Δredβ Δrap
