# Supplementary material for: Temperate Phages Acquire DNA from Defective Prophages by Relaxed Homologous Recombination: The Role of Rad52-Like Recombinases
Source: PLoS Genet. 2014 Mar 6;10(3):e1004181. doi: 10.1371/journal.pgen.1004181 (PMC3945230; doi:10.1371/journal.pgen.1004181)
Supplement: Supplementary file 11 [file pgen.1004181.s006.docx]

FIGURE S6

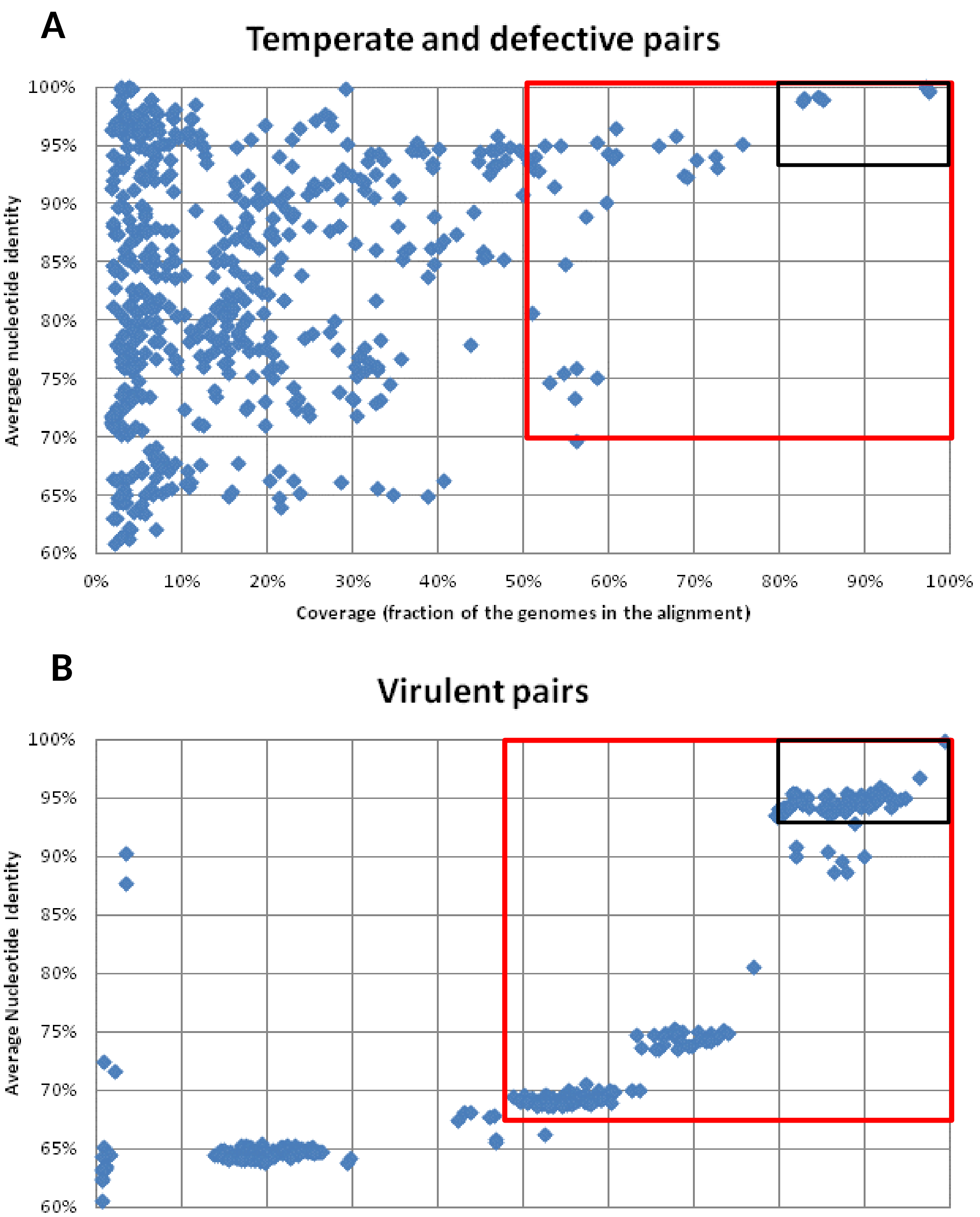
A
Temperate and defective pairs
100%
95%
90%
85%
80%
75%
70%
65%
60%
Average nucleotide identity
0%
10%
20%
30%
40%
50%
60%
70%
80%
90%
100%
Coverage (fraction of the genomes in the alignment)
B
Virulent pairs
100%
95%
90%
85%
80%
75%
70%
65%
60%
Average Nucleotide Identity
